# Sweetened beverage taxes and changes in beverage price, imports and manufacturing: interrupted time series analysis in a middle-income country

**DOI:** 10.1186/s12966-020-00980-1

**Published:** 2020-07-09

**Authors:** Andrea Teng, Viliami Puloka, Murat Genç, Ofeina Filimoehala, Catherine Latu, Mohulamu Lolomana’ia, Sutayut Osornprasop, Louise Signal, Nick Wilson

**Affiliations:** 1 University of Otago Wellington, PO Box 7343, Wellington, New Zealand; 2 University of Otago, Dunedin, New Zealand; 3 Tonga Health Promotion Foundation (TongaHealth), Nuku’alofa, Tonga; 4 Fiji National University, Suva, Fiji; 5 Tonga Customs, Nuku’alofa, Tonga; 6 World Bank, Bangkok, Thailand

**Keywords:** Sugary drinks, Sugar-sweetened beverages, Soft drink, Taxes, Tonga, Pacific, Trade, Evaluation, Quasi experiment, Natural experiment, Time-series

## Abstract

**Background:**

The Pacific Island nation of Tonga (a middle-income country) introduced a sweetened beverage tax of T$0.50/L in 2013, with this increasing further in 2016 (to T$1.00/L), and in 2017 (T$1.50/L; US$0.02/oz). Given the potential importance of such types of fiscal intervention for preventing chronic disease, we aimed to evaluate the impact of these tax changes in Tonga.

**Methods:**

Interrupted time series analysis was used to examine monthly import volumes and quarterly price and manufacturing 1 year after each tax change, compared with a counterfactual based on existing trends. Autocorrelation was adjusted for when present, and adjustments were made for changes in GDP per capita, visitor numbers, season and T$/US$ exchange rate.

**Results:**

In the year after the 2013, 2016 and 2017 tax increases, the price of an indicator soft drink increased by 16.8% (95%CI: 6.3 to 29.6), 3.7% (− 0.6 to 8.3) and 17.6% (6.0 to 32.0) respectively. Imports of sweetened beverages decreased with changes of − 10.4% (− 23.6 to 9.0), − 30.3% (− 38.8 to − 20.5) and − 62.5% (− 73.1 to − 43.4) respectively. Juice imports changed by − 54.2% (− 93.2 to − 1.1), and sachet drinks by − 15.5% (− 67.8 to 88.3) after the 2017 tax increase. Tonga water bottling (T$) increased in value by 143% (69 to 334) after the 2016 tax increase and soft drink manufacturing increased by 20% (2 to 46, albeit 5% market share).

**Conclusions:**

Consistent with international evaluations of sugar-sweetened beverage taxes, the taxes in Tonga were associated with increased prices, decreased taxed beverages imports, and increased locally bottled water.

## Background

There are 14 Pacific Island countries and territories (PICTs) which have introduced sugar-sweetened beverage (SSB) taxes, including at least eight with excise taxes that were introduced since 2000 [1]. Despite the number of SSB taxes in the Pacific there have been few published evaluations from the region [2, 3]. SSB tax evaluations internationally are all from high-income countries (HIC, as classified by the World Bank) with the exception of Mexico, and there has been only one evaluation from a small island developing state (SIDS; Barbados [4], a high-income country) [5]. There is now strong evidence that SSB taxes successfully reduce sales or purchasing of taxed beverages in largely high-income settings [5], but also in other settings (such as Mexico). However, further SSB tax evaluations from different settings are desirable to better understand the impact of context, country income, tax design and substitution to untaxed products. It is possible that low- and middle-income jurisdictions may be more sensitive to price impacts or alternatively that there may be greater challenges in effectively implementing such taxes.

Tonga is a Pacific Island nation, an upper-middle-income country (World Bank), and a Small Island Developing State. The rates of child and adult obesity in Tonga are some of the highest in the world [6–8]. Tonga first introduced an excise tax on sweetened beverages on 13 August 2013 at T$0.50/L [9], replacing an existing 15% import tariff. The change was developed by the Ministry of Revenue and Customs [10] after a directive by then Prime Minister Tuʻivakanō to the Minister of Revenue to work on food, tobacco and alcohol taxes. On 1 July 2016 the tax was doubled to T$1.00/L [11]; and on 27 June 2017 there was a further increase to T$1.50/L; with new changes to broaden the tax to include fruit juice and powdered drink sachets, and specifically target thresholds of sugar concentration [12]. The 2017 tax was applied to sweetened beverages and fruit juices containing > 5 g/100 ml to ≤20 g/100 ml of sugar, ie, the majority of soft drinks, energy drinks and fruit juices with a higher rate of T$4/L for concentrated beverages with > 20 g/100 ml of sugar (see Table 1 for further information). SSB tax revenue totalled T$8.4 million in 2017/18 [3], but it was not earmarked for any specific spending purposes.

**Table 1:** Ad valorem equivalents of the sweetened beverage tax increases by year and beverage category in Tonga

Year	Tax changes	Beverage category	Import unit value (T$/L)	Size of the previous tax (%/AVE)	Size of the new tax (%/AVE)	Size of the tax increase (AVE, % point change)
**2013**	15% tariff → T$0.50/L excise	Sweetened beverages, including flavoured milk HS 22.02	$1.18/L	15% tariff	42%	27%
**2016**	excise T$0.50/L → $1.00/L	Sweetened beverages, including flavoured milk HS 22.02	$1.58/L	32% AVE	63%	32%
**2017**^**a**^	excise T$1.00/L → $1.50/L	Sweetened beverages, including flavoured milk, sugar > 5 g to ≤20 g/100 ml HS 22.02	$1.60/L	62% AVE	94%	31%
	*excise T$1.00/L → $4.00/L*^b^	*Sweetened beverages****high-sugar*** *> 20 g/100 ml* *HS 22.02*	*$1.60/L*	*62% AVE*	*250%*	*188%*
	*excise T$1.00/L → 15% tariff only*^b^	*Sweetened beverages****low-sugar*** *≤ 5 g /100 ml* *HS 20.09*	*$1.60/L*	*62% AVE*	*15% tariff*	*-47% (decrease)*
	15% tariff → $1.50/L excise	Juice, sugar > 5 g to ≤20 g/100 ml HS 20.09	$2.26/L	15% tariff	66%	51%
	*15% tariff → $4.00/L excise*^b^	*Juice****high-sugar*** *> 20 g/100 ml* *HS 20.09*	*$2.26/L*	*15% tariff*	*177%*	*162%*
	*15% tariff remained*^b^	*Juice****low-sugar*** *≤ 5 g /100 ml* *HS 20.09*	*$2.26/L*	*15% tariff*	*15% tariff*	*0%*
	15% tariff → $4/kg excise	Powdered drink sachets HS 1701.91.10	$15.52/kg	15% tariff	26%	11%

Notes: The ad valorem equivalent (AVE) increase is simply the difference between the size of the old tax and the new tax, measured in percentage points. The AVE is calculated as the ratio between the excise tax and the import unit value from Tonga Customs (including cost, insurance and freight and averaged over 1 year spanning the tax change), both measured in T$/L (or T$/kg)

^a^After 2017 only a proportion of the import categories for sweetened beverages and juices were subject to the excise for the first time, with low-sugar beverages exempt and very high sugar beverages subject to a higher excise rate. The legislated > 5 g and ≤ 20 g/100 ml tax changes were used for calculating the 2017 tax changes. This was because the vast majority of sweetened beverages were in the > 5 g to ≤20 g/100 ml of sugar sub category, for example 98% (59/60 items) of SSB container litter collected in a litter survey (October 2018) and 75% (6/8 items) of juice containers

^b^These sub categories accounted for a very small proportion of the import category volumes and were not specifically evaluated in this study

A recent report analysed the impact of taxation policy in Tonga not only on SSBs but also tobacco, alcohol and food products in 2016 and 2017 [3]. The report describes the impact of SSB taxes on surveyed beverage prices and annual import volumes. However, for these measures it did not account for existing price and import trends or the impact of changes in population numbers, economic trends, visitor numbers and exchange rates on study findings. The report included a household survey of consumption behaviours which reported water as the main substitute for taxed beverages with 23% of respondents reporting that they had switched from taxed beverages to water [3]. Local water bottling has grown rapidly in Tonga and this beverage is available and is sold at relatively lower prices, now making up about half of the market share similar to imported products (see Additional file 1: Litter survey). The report also described increased consumption of local juices, coconut water and cheaper locally produced soft drinks.

Other policies and contextual factors may affect the impact of SSB taxes. SSB taxes in Tonga were part of a broader food tax policy initiative on fatty and sugary foods implemented largely in 2016 (chicken quarters, turkey tails, mayonnaise and ice cream) and 2017 (lamb flaps, butter, dairy spreads, sweets, chocolate and biscuits). There was also a tariff exemption on bottled water and milk introduced in July 2016 to replace an existing 15% tariff [13]. During the study period there were no regulations relating to SSB marketing or SSB sales in schools with the exception of a voluntary school food policy which appears to have had little impact [14]. There were no major public awareness campaigns about the SSB tax changes and by 2017 just over one-third of the public were aware of the SSB tax increases [3]. The country is susceptible to tropical cyclones, with substantial destruction to infrastructure from Cyclone Gita on 12 February 2018, with water supply affected in many households. The poverty rate in Tonga also increased after the 2008 global financial crisis with increased cost of imported food and fuel and declines in remittances from abroad [15]. SSBs are widely sold and frequently consumed by adolescents, for example 61% of students reported drinking carbonated soft drinks one or more times per day during the past 30 days, in 2017 [16]. Over half (52%) of food expenditure in Tonga is spent on imported foods, which is high compared to some other Pacific countries [17].

The objective of this study was to examine the impact of SSB tax changes in Tonga on taxed and untaxed beverage (i) prices, (ii) import volumes and (iii) local production (2016 tax only).

## Methods

This study was an interrupted time series design and the protocol is available online: http://hdl.handle.net/10523/9432.

### Datasets

Monthly import data, revenue and tax data were provided by Tonga Customs from the third quarter (Q3) in 2009 to Q2 in 2018. Import volumes were categorised using the International Harmonised System (HS) used for coding international trade flows and assigning beverage tax. Taxed beverage categories included sweetened beverages (HS 2202) such as soft drinks, energy drinks, flavoured milk and artificially-sweetened beverages (untaxed after 2017); fruit and vegetable juice (HS 2009) and powdered drink sachets (HS 1701.91.10) (personal communication Tonga Customs). The juice and sachet categories were only taxed from 2017. For the earlier 2013 and 2016 SSB tax changes, juice and sachet drinks imports were examined as potential substitutes. Imported milk (HS 0401.10 and 0401.20) was examined as a potential substitute in all years, ie, unsweetened milk frequently sold in long-life milk cartons. Imported water (HS 2201) was excluded from the analysis due to the relatively large market share of local water manufacturing (50%, as per protocol). Market share was estimated by comparing import data with Department of Statistics manufacturing reports and from the authors’ litter survey in Tonga in 2018 (see Additional file 1). The majority (95%) of sweetened beverages purchased in Tonga during the study period were imported, thus improving the validity of trends in trade as an estimate of national levels of SSB consumption.

Average prices were provided by the Tonga Department of Statistics from Q4 2010 to Q3 2018 for indicator beverages selected for inclusion in the inflation index; namely 600 ml bottle of Coca-Cola (taxed), 200 ml carton of Zap flavoured milk (taxed), 1 L carton of Golden Circle fruit juice (taxed only in 2017) and a 1 L carton of Anchor long-life milk (untaxed). The Department of Statistics also provided quarterly local manufacturing levels (T$) for soft drinks and bottled water, 2011 census annual population projections, annual gross domestic product (T$ GDP) per capita and monthly visitor numbers. For further information on variable descriptions see Additional file 1: Table A.

### Outcomes

The key outcomes in this study were average price change (T$), per capita import volume (L/person/year) and manufacturing levels (T$/person/year); and each was measured for taxed and untaxed beverages. The absolute difference (rate difference [RD]), percentage change and tax elasticity were estimated. Ad valorem equivalent (AVE) tax rates and tax elasticities (percentage change for each 1 % change in tax) were estimated as outlined in Additional file 1: Tax elasticities. Confidence intervals (95%CI) for RD and % change were boot strapped using Monte Carlo simulation (see Additional file 1: Confidence Intervals).

Import volume outcomes were assessed monthly, a deviation from the quarterly rates planned in the protocol to improve study power. Only quarterly data, however, were available for price and manufacturing outcomes. A 1 year time period of follow-up was selected because this was the maximum period of available data after the 2016 and 2017 taxes, and the tax changes were expected to have become operationalised within the first month. Given interest in the duration of tax effects, study outcomes were also examined in the second year after the 2013 tax and the combined first and second year average.

### Analysis

Interrupted time series analysis was used to examine study outcomes after the 2013, 2016 and 2017 tax changes. The primary comparison was the difference in import volume of taxed beverages compared to the counterfactual based on pre-existing trends. Pre-existing trends were identified from the observed trends in the time-period immediately preceding each tax change (eg, 2009–13, 2013–16 and 2016–17 respectively in the taxed import volume analyses). Generalised least squares (GLS) regression with segmented regression was used to fit a linear model to the data, and errors were allowed to be correlated and/or have unequal variances. Post-tax changes in both the level and trend of import volumes were modelled (see Additional file 1: Analytical model). Autocorrelation and moving averages were assessed by the Durbin-Watson test, graphing the model residuals and by plotting the autocorrelation function and partial autocorrelation function. Model outputs were used to plot predicted and counterfactual outcome trends before and after each tax change. All three SSB tax increases were evaluated in the same model. Potential time-varying confounding was managed by adjusting for trends in per capita GDP (T$), visitor numbers, and season (ie, month in the import analyses or quarter in the other analyses) (as per protocol), with the addition of exchange rate (T$/US$) (whether or not these were significant predictors of the outcome). However, in the manufacturing analysis, season could not be included due to the small number of observations. Sensitivity analyses were carried out to test the use of different model specifications (Additional file 1: Table G). All analyses were done using R (version 3.4.3).

## Results

### Tax changes

The 2013, 2016 and 2017 legislated tax increases on sweetened beverages (including a comparison with the pre-2013 tariff rate) were all similar at 27, 32 and 31% respectively as a proportion of import prices (Table 1). In comparison, the 2017 juice tax was larger (51%), and the 2017 sachet drinks tax was smaller (11%).

### Price results

**Fig. 1:**
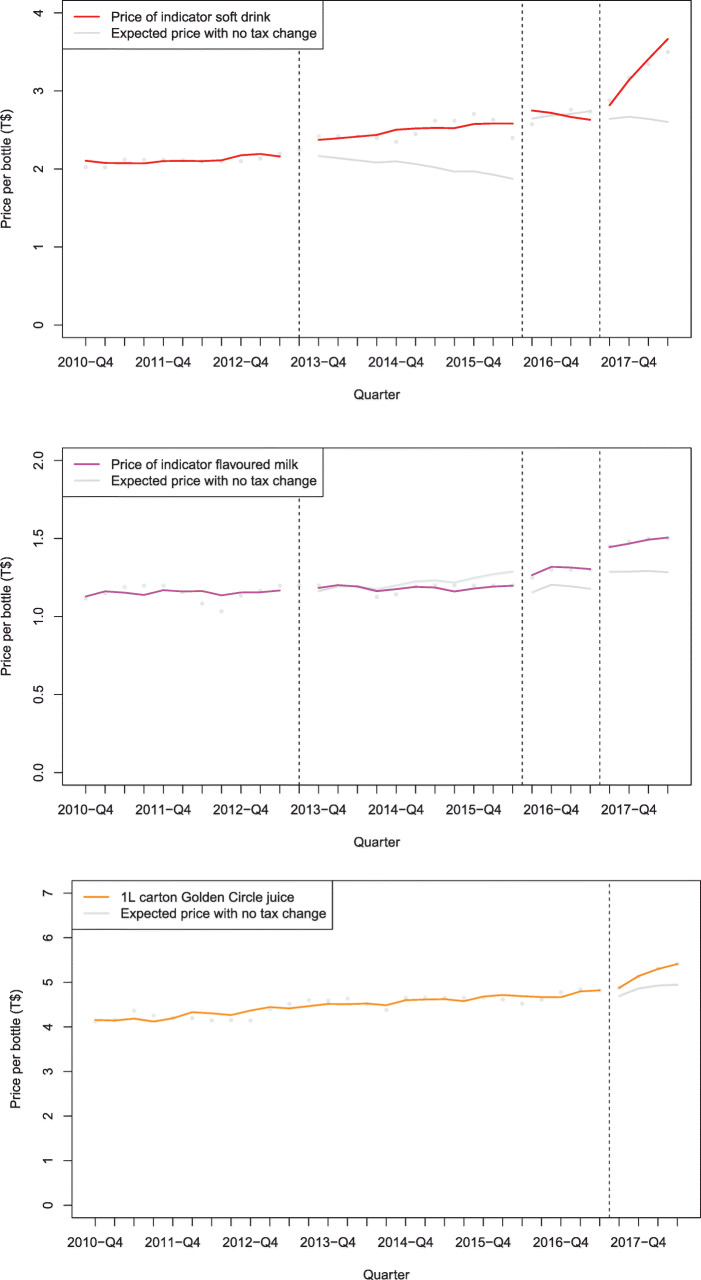
Average price of indicator taxed beverages post SSB tax increases in Tonga in 2013, 2016 and 2017, compared to what was expected based on existing trends, 2009–2018. The SSB tax included soft drinks and flavoured milk in all years, and juice in 2017 only. Notes: Adjusted for autocorrelation, visitors, GDP per capita, season and exchange rate T$/US$. Dashed vertical lines indicate the timing of the tax changes

**Fig. 2:**
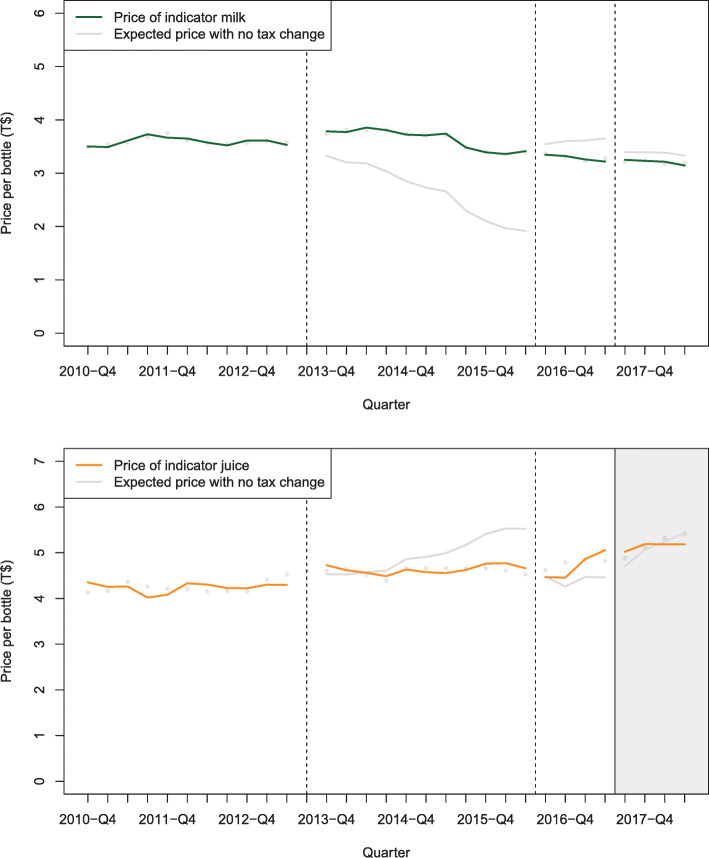
Average price of indicator untaxed beverages post SSB tax increases in Tonga in 2013, 2016 and 2017, compared to what was expected based on existing trends, 2009–2018 (a 15% milk tariff was removed in 2016). Notes: Adjusted for autocorrelation, visitors, GDP per capita, season and exchange rate T$/US$. Vertical lines indicate increases in SSB taxes that did not apply to these beverages. The grey area indicates that juice was less likely to be a potential substitute for taxed beverages because it became included in the tax system

Figure 1 and Additional file 1: Table B describe the impact of SSB taxes on the price of three taxed indicator beverages. In the year after each of the 2013, 2016 and 2017 tax increases, the price of the indicator brand of soft drink increased by 16.8% (6.3 to 29.6), 3.7% (− 0.6 to 8.3), and 17.6% (6.0 to 32.0) respectively compared to the counterfactual. For the indicator flavoured milk these changes were 0.3% (− 11.6 to 15.3), 8.9% (1.4 to 17.6), and 13.7% (2.1 to 28.5) respectively. After being taxed in 2017 the price of the indicator brand of juice increased by 6.7% (2.6 to 10.9). Figure 2 and Additional file 1: Table C summarise the price outcomes for untaxed beverages. In the year after tax increases on sweetened beverages, the average price of indicator milk increased by 19.4% (18.5 to 20.2) for the 2013 tax, and decreased by − 6.1% (− 6.5 to − 5.7) and − 1.9% (− 3.1 to − 0.7) respectively after the 2016 and 2017 tax increases.

### Beverage import results

#### Taxed beverages

**Fig. 3:**
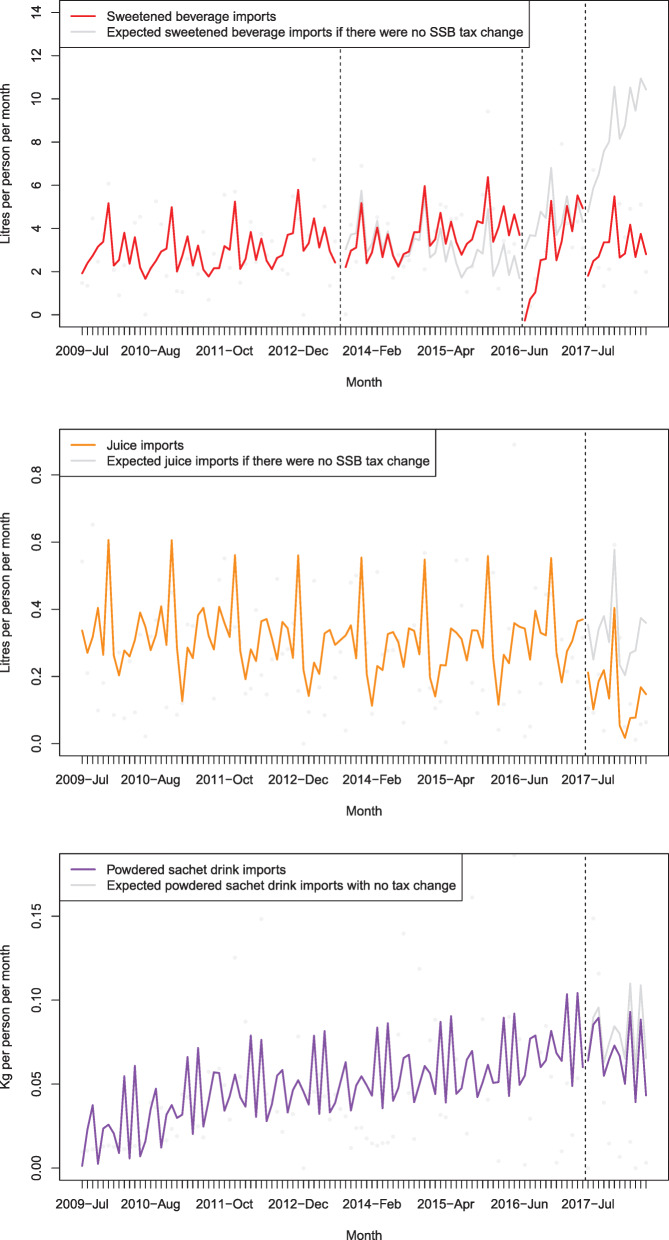
Impact of SSB tax increases in Tonga in 2013, 2016 and 2017 on taxed beverage import volumes, compared to what was expected based on existing trends. Note: Sweetened beverages (Harmonised system [HS] code 2202), juice (HS 2009) and powdered drink sachets (HS 1701.91.10), with adjustment for autocorrelation, GDP per capita, visitor numbers, season and exchange rate (T$/US$), 2009–2018. Source: Tonga Customs

Figure 3 and Additional file 1: Table D present the associated changes in import volumes after the 2013, 2016 and 2017 tax changes for taxed beverages. In the year after the 2013 tax introduction there was a − 10.4% (− 23.6 to 9.0) decline in sweetened beverage import volumes compared to the counterfactual. After the 2016 tax this was − 30.3% (− 38.8 to − 20.5) and − 62.5% (− 73.1 to − 43.4) after the 2017 tax. The equivalent change in import volumes for each 1% increase in tax (estimated elasticity) were − 0.38 (− 0.86 to 0.33), − 0.96 (− 1.22 to − 0.65) and − 2.00 (− 2.34 to − 1.39), following the 2013, 2016 and 2017 tax changes respectively. The 2013 decline in sweetened beverage volumes appeared to only persist for the first year. In the second year after the tax change, import volumes were 21.9% (− 22.4 to 167.6) greater than what was expected compared to the counterfactual. After the 2017 tax, taxed juice import volumes decreased with a change of − 54.2% (− 93.2 to − 1.1; estimated elasticity: − 1.06) and taxed sachet drinks decreased with a change of − 15.5% (− 67.8 to 88.3; estimated elasticity: − 1.44).

#### Untaxed beverages

**Fig. 4:**
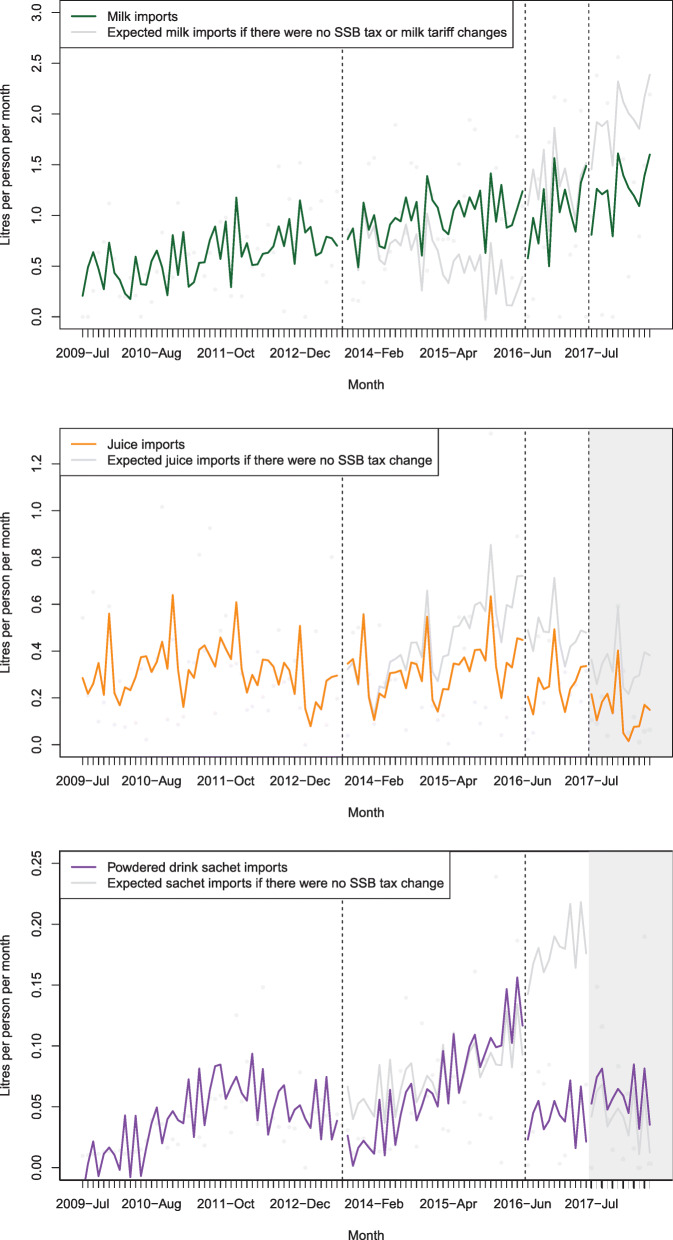
Impact of SSB tax increase in Tonga in 2013, 2016 and 2017 on untaxed beverage import volumes, compared to what was expected based on existing trends (the 15% tariff on milk was removed in 2016). Note: Milk (HS 0401.10 and 0401.20), juice (HS 2009) and powdered drink sachets (HS 1701.91.10), with adjustment for autocorrelation, GDP per capita, visitor numbers, season and exchange rate T$/US$, 2009–2018. The dashed line denotes the timing of the SSB tax changes. The grey area indicates a period when the beverage was taxed and substitution from the other taxed beverages was less likely to be occurring. Note a 15% import tariff was removed from milk (tax decrease) at the time of the 2016 tax change, but otherwise these beverages were not subject to any tax changes. Source: Tonga Customs

Figure 4 and Additional file 1: Table E outline the association between beverage taxes and the corresponding import volumes of untaxed beverages. There was no evidence of any significant substitution to milk, juice or powdered drink sachets. There was an increase in import volume of milk 1 year after the 2013 tax of 16.6% (− 11.5 to 70.0), but declines after the tax increases in 2016 and 2017 of − 20.9% (− 33.4 to − 4.9) and − 36.6% (− 54.9 to 1.2) respectively. The declines in sweetened beverage import volumes in each year were all greater than the corresponding declines in milk imports.

### Local manufacturing results

**Fig. 5:**
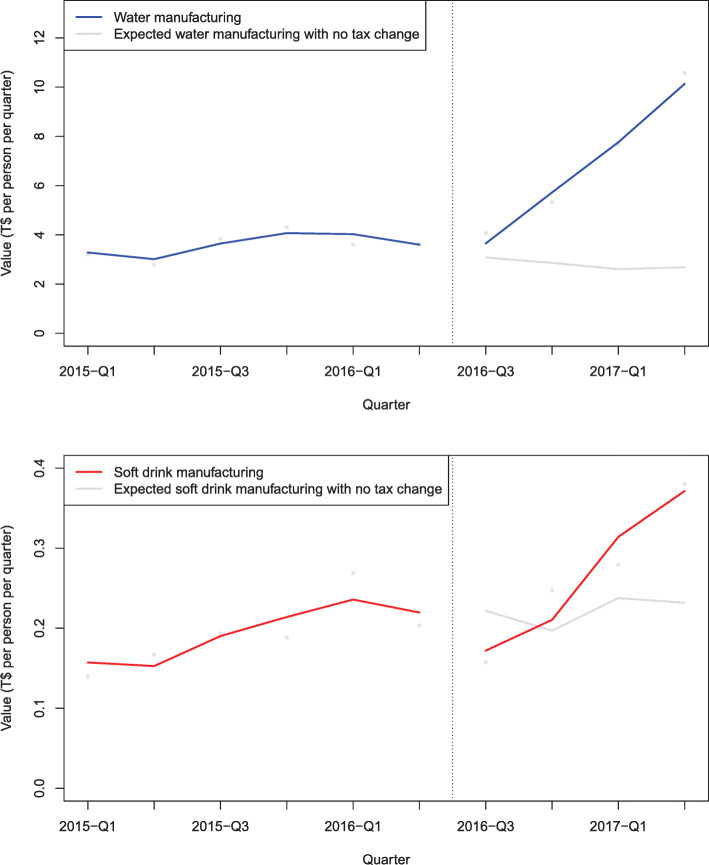
Water and soft drink manufacturing value in Tonga in the first year after the 2016 sweetened beverage tax increase. Notes: Tax change was from T$0.50 to T$1.00/L. Comparison is with what was expected based on existing trends, adjusted for visitor numbers, GDP per capita, exchange rate T$/US$ and autocorrelation

Local manufacturing of water and soft drinks in Tonga increased over time (Fig. 5, Additional file 1: Table F). The 2016 tax change was associated with an increase in the value of soft drink manufacturing (20%, CI: 2 to 46%, albeit with an estimate 5% market share) and an increase in the value of water manufacturing (143%, CI: 69 to 334%) both compared to the counterfactual. The 2016/17 value of soft drink manufacturing was T$1.07/person, and bottled water manufacturing was T$27.27/person.

## Discussion

### Main findings and interpretation

The three tax increases on sweetened beverages were all approximately 30%, with larger increases on juice and smaller increases on sachet drinks in 2017. There were consistent trends of price increases for all the taxed beverage products in Tonga in the year after each of the tax increases, which were generally statistically significant. These were in the same direction as previous retail survey findings comparing before and after the 2016 and 2017 taxes (increases of 14 and 4% respectively) [3]. Taxed beverage import volumes consistently declined compared to the counterfactual in the year after tax changes in 2013, 2016, and 2017, by − 10, − 30% and − 63% respectively for sweetened beverages (with estimated elasticities of: − 0.38, − 0.96, − 2.00), and in 2017 by − 54% for juice (estimated elasticity: − 1.06) and − 16% for sachet drinks (estimated elasticity: − 1.44), and most were statistically significant. These were larger declines than what was previously described in Tonga (− 19% decrease and 16% increase in sweetened beverages after the 2016 and 2017 taxes respectively) [3], likely because the counterfactual in this comparison was based on background import trends. Price and import findings were consistent with economic theory whereby price increases reduce demand and thereby reduce import volumes of goods that are substantially imported. If there had been a public awareness campaign, the impact of Tongan beverage taxes may have been even greater [18].

Import changes were on average proportional to the size of the tax changes (estimated elasticities around: − 1.0), and similar to previous elasticity estimates from a meta-analysis of evaluations from largely high-income jurisdictions [5]. Average estimated elasticities for sweetened beverage taxes in Tonga were similar to those reported for a SSB tax in Mexico, but less than those for SSB taxes in Chile, France, and Catalonia; and greater than those reported for the small island state of Barbados (elasticity: − 0.4, with a − 4.3% reduction in grocery sales after a 10% ad valorem tax) [4], Berkeley (California, US; evidence of cross border shopping), and earlier US taxes (smaller sales taxes which may not be displayed). There was no evidence that the extent of the response to SSB taxes in Tonga or price sensitivity differed from high-income countries. However, estimated elasticities in Tonga differed between tax changes, with smaller decreases in sweetened beverage import volumes in 2013 and greater declines in 2017.

It was unclear exactly why the impact of the 2013 tax appeared to be less and the 2017 tax much greater, but changes in context and tax design may have been important. In 2016 and 2017 the application of excise to a range of fatty and sugary foods [3], is likely to have reduced the available household grocery budget and may have limited discretionary spending on sugary drinks in those years. Greater expansion of local bottled water production from 2015 onwards appears to have provided a cheap and widely available healthy alternative to taxed beverages that may have increased the impact of the 2016 and 2017 taxes.

The improved threshold-based tax design in 2017 may have influenced the larger decrease in SSB import volumes after the 2017 tax compared to after the earlier volumetric SSB tax designs. The tax targeted a broader range of SSBs and provided a price incentive for importing more low-sugar beverages, possibly mirroring how threshold taxes have encouraged reformulation in larger economies such as the UK [19]. The 2017 sweetened beverage decline may also have been overestimated by the steep trend in the counterfactual. The decline was only half the size when this trend change was removed from the model (Additional file 1: Table G).

A longer period of follow-up was examined after the 2013 sweetened beverage excise. In the second year there were greater taxed beverage import volumes than the counterfactual, possibly influenced by an increase in cheaply available imported sweetened beverages eg, from Malaysia (UN Comtrade), recovering inflows of remittance payments after the global financial crisis or other external factors. Findings for Tonga contrast with the sustained response in Mexico after 2 years [20].

There was evidence of substitution to locally manufactured soft drinks and bottled water after the 2016 tax increase in Tonga. The 20% increase in soft drink manufacturing in Tonga may have been due to the much cheaper prices of local drinks making them more attractive compared to the increasingly expensive imported beverages. Although legislated since 2013, SSB excise revenue collection did not start for locally manufactured products until as recently as 2019. However, the market share of manufactured soft drinks was likely small (5%), suggesting this shift would have had only a small effect on overall consumption.

The very large 143% increase in the value of bottled water manufacturing, was consistent with the purchasing patterns from other jurisdictions with SSB taxes [5, 21, 22], but was of a larger magnitude. For example, after the 2015 Barbados tax change of 10%, sales of bottled water increased by 7.5% (95%CI: 6.5 to 8.3%; cross-price elasticity: + 0.75) and there was a move to cheaper beverages [4]. In Tonga, there was no evidence of any substitution to untaxed beverage imports (untaxed milk, juice and sachet drinks) which instead tended to decrease with each tax change.

### Study strengths and limitations

To the best of our knowledge, this is the first evaluation in the scientific journal literature of the impact of a SSB tax in a Pacific Island jurisdiction. Similarly, it is only the second such evaluation in a middle-income country (after Mexico) and second for a Small Island Developing State (ie, after Barbados). This study expands on the recent Tonga tax evaluation [3] by reporting statistical significance, allowing for existing trends, adjusting for multiple confounders and autocorrelation, and using monthly import data to improve study power. The combination here of price, import volumes and manufacturing outcomes, for taxed and untaxed beverages, extends our understanding of the wider impact of SSB taxes. Import volumes represent national levels of beverage consumption purchased from any location, only possible given the low market share of locally produced SSBs.

Imports however, do not fully equate to dietary intake because there may be potential stockpiling and wasted products. Furthermore, there are other beverages not included in this analysis such as nu (‘drinking coconuts’) and otai (a smoothie from watermelon, pineapple and coconut milk) sold in markets; and beverages such as hot drinks and tap water. Price data were only available for four indicator beverages, which may not fully represent price changes in overall beverage categories, for example in other brands of soft drinks. Manufacturing outcomes spanned the 2016 tax change only and were only measured in value rather than volume. Finally, the study was limited to whole population data and could not examine outcomes within subsets of the population.

There may be unmeasured and residual time-varying confounding that was not accounted for and confounding from month to month changes in GDP and population levels for which we only had annual trends. Other policies and economic changes may have affected study results such as the food taxes discussed above, macroeconomic changes or weather patterns. Such a factor might have contributed to a more general import volume decline across taxed and untaxed beverages. The lack of an appropriate geographical comparison country reduced the ability to control for external factors experienced by both countries.

The power in this study was improved by a combination of the large expected effect of tax changes of 11 to 51%, 12+ data points [23], before and after each tax change in the import analyses, and limited point-to-point variability [24] in the price and manufacturing analyses. Price and manufacturing comparisons relying on four quarterly data points before and after the tax change were expected to have limited power, but several statistically significant trends were still identified, suggesting a recommended minimum of 9+ data points [25] was not necessary, although further data points may reduce the influence of any outliers.

### Implications

Moderate sized SSB tax increases in this middle-income country on a relatively comprehensive set of taxed beverages successfully reduced import volumes of taxed beverages and contributed to government revenue. The findings are likely to be broadly generalizable to other jurisdictions, and indeed they were broadly similar to what has been found in other studies. Ongoing evaluation of SSB taxes is desirable in all settings to better understand their impact (for example on SSB consumption, substitution and potential health benefits), which may be affected by context and study design and differ over time. Synergistic effects with other NCD policies such as media campaigns, health warning labels and food taxes could be considered. Excise rates on locally produced and imported beverages should ideally be equal and fully implemented to limit trends towards unhealthy substitution. Jurisdictions can also consider ongoing SSB tax design developments, for example taxing the sugar content of beverages [26] and designing taxes which stimulate more imports of low-sugar beverages. Increased investment of tax revenue into health and obesity prevention may be particularly beneficial in Tonga and other countries with serious epidemics of obesity. For settings with limited reticulated safe drinking water supplies (such as Tonga), there is a case for further government investment in improving such supplies, to ensure that healthy substitutes to sweetened beverages are readily available – especially in schools and for citizens with the lowest incomes. Revenue from SSB taxes can potentially be used for such purposes and for improving nutrition in other ways such as healthy food in schools.

## Conclusion

Consistent with international findings on the impact of SSB taxes, the taxes in Tonga were associated with increased prices, decreased taxed beverage imports, increased locally bottled water, and more concerning also a small increase in the local manufacturing of soft drinks.

## Supplementary information

**Additional file 1.** Appendices (Additional Background, Methods and Results).

**Additional file 2.** Study protocol, also available online at http://hdl.handle.net/10523/9432.

**Additional file 3.** STROBE-nut Checklist: An extension of the STROBE statement for nutritional epidemiology.

## Data Availability

The data that support the findings of this study are available from Tonga Customs and Tonga Department of Statistics but restrictions apply to the availability of these data, which were used after Tonga research permit approval in the current study, and so are not publicly available. Data are however available from the authors upon reasonable request and with permission of Tonga Customs and Tonga Department of Statistics.

## Abbreviations

GDP: Gross domestic product
NCD: Non-communicable disease
SSB: Sugar-sweetened beverage
