# Supplementary material for: Sweetened beverage taxes and changes in beverage price, imports and manufacturing: interrupted time series analysis in a middle-income country
Source: Int J Behav Nutr Phys Act. 2020 Jul 9;17:90. doi: 10.1186/s12966-020-00980-1 (PMC7350205; doi:10.1186/s12966-020-00980-1)
Supplement: Supplementary file 1 — Additional file 1. Appendices (Additional Background, Methods and Results). [file 12966_2020_980_MOESM1_ESM.docx]

# Additional file 1: Appendices

Contents

[Further methods 2](#_Toc36458475)

[Data sources and variable descriptions 2](#_Toc36458476)

[Tax pass-through could not be calculated 3](#_Toc36458477)

[Tax elasticities 3](#_Toc36458478)

[Confidence intervals 3](#_Toc36458479)

[Analytical model 3](#_Toc36458480)

[Further results 4](#_Toc36458481)

[Further results corresponding to Figures 1 to 5 4](#_Toc36458482)

[Sensitivity tests 9](#_Toc36458483)

[Modelling the counterfactual 12](#_Toc36458484)

[Litter survey 13](#_Toc36458485)

[Methods 13](#_Toc36458486)

[Results of litter distribution and origin 18](#_Toc36458487)

[Market share of locally produced soft drinks 18](#_Toc36458488)

[References 18](#_Toc36458489)

## Further methods

### Data sources and variable descriptions

**Table A: Data sources and variable descriptions**

| **Cook Islands** | **Definition** | **Data source** | **Frequency** | **Timeline** | **Any adjustments** |
| --- | --- | --- | --- | --- | --- |
| Trade volume | Volume of beverage category imported into Tonga (L) | Tonga Customs (Mohulamu) | Monthly | 2009 to 2018 | Summed to give quarterly trade volumes |
| Trade value | Cost, insurance and freight (CIF) value for each beverage category (T$) | Tonga Customs (Mohulamu) | Monthly | 2009 to 2018 | Summed to give quarterly trade values |
| Trade revenue | Revenue sub-grouped into import duty, excise and consumer tax | Tonga Customs (Mohulamu) | Monthly | 2009 to 2018 | Summed to give quarterly revenue figures |
| Price of specific beverages | 600ml Coca-Cola soft drink, based on data from 4 outlets every quarter (will be 7 from Q4 2018).  200ml carton of Zap flavoured milk, based on data from 4 outlets every quarter (no longer collected from Q4 2018).  1L carton of Golden Circle juice, based on data from 4 outlets every quarter (no longer collected from Q4 2018).  1L Anchor long-life milk, based on data from 11 outlets every quarter (will be 12 from Q4 2018). | Tonga Department of Statistics (Ofa Takai) | Quarterly average prices | Q4 2010 to Q3 2018 |  |
| Manufacturing level | Value of manufacturing (T$) available for water and soft drinks | Tonga Department of Statistics (Ilaisaane Tuipulotu) | Quarterly | Start of 2015 to mid-2017 |  |
| Resident population | Total population projections based on the 2011 Tonga census. Note 2016 census showed that the population had decreased faster than expected, however these projections are were not available at the time of writing. | Tonga Department of Statistics (Sione Lolohea) | Quarterly, midpoint | 2010 to 2018 | Data was extrapolated available data backwards two quarters using the same projections.  Based on midyear population estimates from 2011 to 2018.  Smoothed to monthly using modelled quadratic equation. |
| Gross domestic product | Annual total gross domestic product per capita, at constant T$ 2010/11 prices, factor cost (excluding taxes/subsidies). Reported in thousands. | Tonga Department of Statistics (Masiva Masila) | Reported for each financial year | 2007/08 to 2017/18 | Smoothed by averaging across months.  Converted into GDP per capita using population estimates. |
| Visitor numbers | All arrivals to Tonga excluding returning residents. Includes air, yacht and ship arrivals. | Tonga Department of Statistics (Salote Latu) | Monthly | Q1 2002 to Q4 2018 | Summed to quarterly as required. |
| Season | Dummy variable 1,2,3,4 for each quarter in a calendar year in price and manufacturing analyses, or for each month in the import analyses. | Dummy variable | Monthly or Quarterly | any | Season was changed to month with 12 different values for the monthly data sensitivity analysis |
| Exchange rate | Local currency unity (LCU) per USD  market rate, period average (calendar year) | Fxtop foreign historical exchange rates  <https://fxtop.com/en/historical-exchange-rates.php?A=1&C1=USD&C2=TOP&MA=1&DD1=01&MM1=01&YYYY1=2009&B=1&P=&I=1&DD2=30&MM2=06&YYYY2=2019&btnOK=Go%21> | Monthly average exchange rates | 2009 to 2018 | Averaged to quarterly as required. |
| Cyclone | Cyclone Gita, Tongatapu Q1 2018 and Cyclone Ian, H’apai Q1 2014 each modelled with separate dummy variables for the month/two months affected | Wikipedia | One-off | 2014 and 2018 |  |

### Tax pass-through could not be calculated

Tax elasticities were not able to be used to estimate tax pass-through of taxed beverages in the price time series analyses, ie, the proportion of the tax that is passed through to shelf price. This was because the ad valorem equivalent (AVE) level of SSB tax may be a large overestimate of tax size to the extent that shelf prices were larger than import prices. This would severely underestimate tax elasticity and tax pass-through measures. No other longitudinal data on shelf prices were available.

### Tax elasticities

Tax elasticity was defined as the percentage change in import volumes for each 1% increase in the ad valorem equivalent tax rate in the first year after the tax change. Tax elasticities enable study outcomes to be better compared between different sized tax changes. To calculate elasticities, the size of the volumetric tax changes (ie, applied per litre of sweetened beverage) were converted into AVEs. This was done using Tonga Customs trade unit values (T$/L) in the year of tax introduction. This method has been used in meta-analyses [1] and in trade databases [2]. 2017 taxes were calculated based on the >5g to ≤20g/100ml of sugar sub-category because the majority of sweetened beverages had this concentration of sugar in a litter survey (October 2018), for example 98% (59/60 items) of SSB container litter and 75% (6/8 items) of juice containers.

We note that this method of tax elasticity calculation may over estimate the size of the tax as a proportion of shelf price, because import prices are likely to be less than shelf prices. Shelf prices were not available at all time points.

### Confidence intervals

Confidence intervals for each study outcome were calculated using Monte Carlo simulation. The distribution of model coefficients and their standard errors were randomly sampled from 100,000 times, to calculate 100,000 predicted and counterfactual values. For each simulation the absolute differences and percentage change outcomes were calculated as an average across the time period of interest (for example the first year). The 2.5^th^ and the 97.5^th^ percentile of these 100,000 outcomes provided the upper and lower limits for a 95% confidence interval (CI). CIs for tax elasticity outcomes were calculated from the upper and lower limits of the confidence interval for the percentage change.

### Analytical model

This analysis was possible because of the sudden expected impact of a tax on price and import volumes [3] and the tax changes were large enough to signal an expected detectable effect. The ITS design allowed us to control for secular trends in the data, provide a clear graphical presentation of results and adjust for autocorrelation and time-varying confounding (season as a categorical variable, GDP and visitor numbers). [3, 4]

The following equation provides an example of the fitted linear time series regression model.

$$Import volume \left( L/{population} \right)=\beta_{0} + \beta_{1}.{time}_{t}+ \beta_{2}.{tax13}_{t}+ \beta_{3}.{trend13}_{t}+ \beta_{4}.{tax16}_{t}+ \beta_{5}.{trend16}_{t}+ \beta_{6}.{tax17}_{t}+ \beta_{7}.{trend17}_{t}+ \beta_{8}.{GDP}_{t}+ \beta_{9}.{visitors}_{t}+ \beta_{10}.{season}_{t}+ \in_{t}$$

(1)

Key; β_0_ was the intercept, β_1_ was the pre-tax trend, β_2_ was the level change after the 2013 tax, β_3_ was the trend change after the 2013 tax, β_4_ was the level change after the 2016 tax, β_5_ was the trend change after the 2016 tax, β_6_ was the level change after the 2017 tax, β_7_ was the trend change after the 2017 tax, β_8_ was the association between GDP per capita and outcome, β_9_ was the association between visitor numbers and outcome, β_10_ was the association between season and outcome, and ∈ was the error term.

Autocorrelation and moving averages were assessed by the Durbin-Watson test, graphing the model residuals and by plotting the autocorrelation function and partial autocorrelation function. Autocorrelation adjusted models and models without adjustment for autocorrelation were compared using a log-likelihood ratio test. If the autocorrelated model was significantly different from the unadjusted model and had a better fit (lower AIC) then the autocorrelated model was selected, otherwise the model with no autocorrelation was selected as the final model.

## Further results

### Further results corresponding to Figures 1 to 5

**Table B: Changes in the average quarterly price (T$ per item) of taxed beverages in the first and second year post introduction of sweetened beverage taxes in Tonga, time series analysis 2010-2018**

| **Tax change** | **Beverage category** | **Follow-up** | **Tax change (AVE)** | **Observed** | **Counter-factual** | **Absolute difference**  **T$ (95% CI)** | **Percentage change**  **(95% CI)** |
| --- | --- | --- | --- | --- | --- | --- | --- |
| TAXED BEVERAGES | |  |  |  |  |  |  |
| **2013**  15% tariff increased to T$0.50/L excise | Coca-Cola 600ml | First year | 27% | 2.40 | 2.05 | 0.34 (0.14 to 0.55) | 16.8% (6.3 to 29.6) |
|  |  | Second year |  | 2.53 | 1.83 | 0.69 (0.12 to 1.27) | 37.7% (5.0 to 99.1) |
|  |  | Combined |  | 2.46 | 1.94 | 0.52 (0.14 to 0.90) | 26.7% (6.0 to 57.6) |
|  | Zap flavoured milk 200ml | First year | 27% | 1.18 | 1.18 | 0.00 (-0.15 to 0.16) | 0.3% (-11.6 to 15.3) |
|  |  | Second year |  | 1.18 | 1.22 | -0.04 (-0.37 to 0.29) | -3.4% (-24.5 to 32.1) |
|  |  | Combined |  | 1.18 | 1.20 | -0.02 (-0.26 to 0.22) | -1.6% (-18.2 to 22.5) |
| **2016**  excise increased from T$0.50/L to $1.00/L | Coca-Cola 600ml | First year | 32% | 2.72 | 2.62 | 0.10 (-0.02 to 0.21) | 3.7% (-0.6 to 8.3) |
|  | Zap flavoured milk 200ml | First year | 32% | 1.30 | 1.19 | 0.11 (0.02 to 0.20) | 8.9% (1.4 to 17.6) |
| **2017**  excise increased from T$1.00/L to $1.50/L | Coca-Cola 600ml | First year | 31% | 3.22 | 2.74 | 0.48 (0.18 to 0.79) | 17.6% (6.0 to 32.0) |
|  | Zap flavoured milk 200ml | First year | 31% | 1.48 | 1.30 | 0.18 (0.03 to 0.33) | 13.7% (2.1 to 28.5) |
|  | Golden Circle juice 1L | First year | 51% | 5.18 | 4.86 | 0.32 (0.13 to 0.52) | 6.7% (2.6 to 10.9) |

Notes: Observed (Obs), Counterfactual (cfac) is what was expected based on existing trends, Ad valorem equivalent (AVE) is calculated by applying excise rates(T$/L) to import unit values (T$/L). Data kindly provided by Tonga Department of Statistics. Time series difference estimates were adjusted for autocorrelation, visitors, GDP per capita, season and exchange rate T$/US$.

**Table C: Changes in the average quarterly price (T$ per item) of untaxed beverages in the first and second year post introduction of sweetened beverage tax increases in Tonga, time series analysis 2010-2018**

| **Tax change** | **Beverage category** | **Follow-up** | **Tax change (AVE)** | **Observed** | **Counter-factual** | **Absolute difference**  **T$ (95% CI)** | **Percentage change**  **(95% CI)** |
| --- | --- | --- | --- | --- | --- | --- | --- |
| UNTAXED BEVERAGES | |  |  |  |  |  |  |
| **2013**  15% tariff remained | Anchor long-life milk 1L | First year | 0% | 3.81 | 3.19 | 0.62 (0.59 to 0.64) | 19.4% (18.5 to 20.2) |
|  |  | Second year |  | 3.66 | 2.63 | 1.03 (0.98 to 1.08) | 39.1% (36.4 to 41.9) |
|  |  | Combined |  | 3.73 | 2.91 | 0.82 (0.79 to 0.86) | 28.3% (26.6 to 30.0) |
| 15% tariff remained | Golden Circle juice 1L | First year | 0% | 4.60 | 4.56 | 0.04 (-0.08 to 0.16) | 0.9% (-1.7 to 3.5) |
|  |  | Second year |  | 4.60 | 4.98 | -0.39 (-0.75 to -0.02) | -7.7% (-14.0 to -0.5) |
|  |  | Combined |  | 4.60 | 4.77 | -0.17 (-0.41 to 0.07) | -3.6% (-8.2 to 1.5) |
| **2016**  15% tariff decreased to 0% | Anchor long-life milk 1L | First year | -15%* | 3.29 | 3.50 | -0.21 (-0.23 to -0.20) | -6.1% (-6.5 to -5.7) |
| 15% tariff remained | Golden Circle juice 1L | First year | 0% | 4.71 | 4.52 | 0.19 (0.12 to 0.26) | 4.1% (2.5 to 5.8) |
| **2017**  0% tariff remained | Anchor long-life milk 1L | First year | 0% | 3.21 | 3.27 | -0.06 (-0.10 to -0.02) | -1.9% (-3.1 to -0.7) |

Notes: Observed (Obs), Counterfactual (cfac) is what was expected based on existing trends, Ad valorem equivalent (AVE) is calculated by applying excise rates(T$/L) to import unit values (T$/L). Data kindly provided by Tonga Department of Statistics. Time series difference estimates were adjusted for autocorrelation, visitor numbers, GDP per capita, season and exchange rate T$/US$. * A 15% tariff on milk was removed in 2016.

**Table D: Impact of SSB tax increases on taxed beverage import volumes (litres a year per population), Tonga 2009-2018**

| **Tax date** | **Tax increases** | **Beverage category** | **Follow-up** | **Observed (L/p/yr)** | **Counter-factual (L/p/yr)** | **Absolute difference**  **L/p/yr (95% CI)** | **Percentage change**  **(95% CI)** | **Estimated elasticity** |
| --- | --- | --- | --- | --- | --- | --- | --- | --- |
| 13 August  **2013** | 15% tariff → T$0.50/L excise | Sweetened beverages, includes flavoured milk  HS 22.02 | First year | 37.0 | 41.3 | -4.3 (-11.7 to 3.0) | -10.4% (-23.6 to 9.0) | -0.38 (-0.86 to 0.33) |
|  |  |  | Second year | 45.0 | 36.9 | 8.1 (-12.7 to 28.7) | 21.9% (-22.4 to 167.6) | 0.69 (-0.71 to 5.29) |
|  |  |  | Combined | 41.0 | 39.1 | 1.9 (-12.1 to 15.7) | 4.9% (-22.7 to 62.8) | 0.16 (-0.73 to 2.01) |
| 1 July **2016** | excise T$0.50/L →$1.00/L | Sweetened beverages, includes flavoured milk  HS 22.02 | First year | 37.2 | 53.4 | -16.2 (-22.1 to -10.1) | -30.3% (-38.8 to -20.5) | -0.96 (-1.22 to -0.65) |
| 1 July **2017** | excise T$1.00/L →$1.50/L if sugar  >5g to ≤20g/100ml | Sweetened beverages, includes flavoured milk  HS 22.02 | First year | 38.1 | 101.6 | -63.5 (-95.9 to -31.2) | -62.5% (-73.1 to -43.4) | -2.00 (-2.34 to -1.39) |
|  | 15% tariff → $1.50/L excise if sugar >5g to ≤20g/100ml | Juice  HS 20.09 | First year | 1.8 | 3.9 | -2.1 (-4.2 to 0.0) | -54.2% (-93.2 to -1.1) | -1.06 (-1.82 to -0.02) |
|  | 15% tariff → $4/kg excise | Powdered drink sachets  HS 1701.91.10 | First year | 0.8 | 1.0 | -0.1 (-0.8 to 0.5) | -15.5% (-67.8 to 88.3) | -1.44 (-6.30 to 8.20) |

Note: Results are calculated from a GLS model, with Monte Carlo simulation of model outputs and their errors to calculate 95% confidence intervals for the rate difference (RD) and the percentage change (%) compared to baseline. Adjusted for autocorrelation, visitor numbers, GDP per capita, season and exchange rate T$/US$.

**Table E: Impact of SSB taxes on untaxed beverage import volumes (litres a year per person) to test for potential substitution, Tonga 2009-2018**

| **Tax date** | **Tax level** | **Beverage category** | **Follow-up** | **Observed (L/p/yr)** | **Counter-factual (L/p/yr)** | **Absolute difference**  **L/p/yr (95% CI)** | **Percentage change**  **(95% CI)** | **Estimated cross price elasticity** |
| --- | --- | --- | --- | --- | --- | --- | --- | --- |
| 13 August  **2013** | 15% tariff remained | Milk  HS 0401.10 & 0401.20 | First year | 10.5 | 9.0 | 1.5 (-1.4 to 4.3) | 16.6% (-11.5 to 70.0) | 0.60 (-0.42 to 2.55) |
|  |  |  | Second year | 12.3 | 7.1 | 5.2 (-2.1 to 12.6) | 73.7% (-31.9 to 973.1) | 2.68 (-1.16 to 35.41) |
|  |  |  | Combined | 11.4 | 8.1 | 3.4 (-1.6 to 8.3) | 41.7% (-12.7 to 265.9) | 1.52 (-0.46 to 9.68) |
|  | 15% tariff remained | Juice  HS 20.09 | First year | 3.4 | 3.7 | -0.3 (-3.5 to 2.9) | -8.4% (-59.0 to 269.8) | -0.30 (-2.15 to 9.82) |
|  |  |  | Second year | 3.7 | 5.3 | -1.6 (-8.4 to 5.2) | -30.3% (-517.8 to 502.9) | -1.10 (-18.84 to 18.30) |
|  |  |  | Combined | 3.6 | 4.5 | -1.0 (-5.8 to 3.9) | -21.2% (-262.2 to 425.2) | -0.77 (-9.54 to 15.47) |
|  | 15% tariff remained | Powdered drink sachets  HS 1701.91.10 | First year | 0.4 | 0.7 | -0.3 (-1.0 to 0.2) | -49.8% (-90.5 to 105.7) | -1.81 (-3.29 to 3.85) |
|  |  |  | Second year | 0.8 | 0.9 | -0.1 (-1.4 to 1.2) | -8.2% (-765.8 to 777.4) | -0.30 (-27.87 to 28.29) |
|  |  |  | Combined | 0.6 | 0.8 | -0.2 (-1.1 to 0.7) | -26.3% (-347.8 to 430.6) | -0.96 (-12.66 to 15.67) |
| 1 July **2016** | tariff 15% -> 0% | Milk  HS 0401.10 & 0401.20 | First year | 12.6 | 15.9 | -3.3 (-6.0 to -0.7) | -20.9% (-33.4 to -4.9) | -0.66 (-1.05 to -0.15) |
|  | 15% tariff remained | Juice  HS 20.09 | First year | 3.1 | 5.7 | -2.6 (-5.7 to 0.6) | -44.8% (-74.2 to 19.1) | -1.41 (-2.34 to 0.60) |
|  | 15% tariff remained | Powdered drink sachets  HS 1701.91.10 | First year | 0.5 | 2.1 | -1.6 (-2.2 to -1.0) | -76.5% (-90.0 to -60.0) | -2.41 (-2.84 to -1.89) |
| 1 July **2017** | 0% tariff remained | Milk  HS 0401.10 & 0401.20 | First year | 14.9 | 23.5 | -8.6 (-17.3 to 0.2) | -36.6% (-54.9 to 1.2) | -1.17 (-1.76 to 0.04) |

Note: Results are calculated from a GLS model, with Monte Carlo simulation of model outputs and their errors to calculate 95% confidence intervals for the rate difference (RD) and the percentage change (%) compared to baseline. Results were adjusted for autocorrelation, visitor numbers, GDP per capita, season and exchange rate T$/US$. Note a 15% import tariff was removed (a tax decrease) from milk at the time of the 2016 tax change.

**Table F: Manufacturing of soft drinks and bottled water per population in the first post-2016 SSB tax change in Tonga compared to what was expected based on existing trends, manufacturing value (T$ per population per year)**

| **Year** | **Tax change** | **Beverage** | **Observed**  **T$/p/year** | **Counter-factual**  **T$/p/year** | **Absolute difference**  **T$/p/year (95% CI)** | **Percentage change**  **(95% CI)** | **Elasticity** |
| --- | --- | --- | --- | --- | --- | --- | --- |
| 2016 | excise T$0.50/L →$1.00/L | Soft drinks, taxed | 1.07 | 0.89 | 0.18 (0.02 to 0.34) | 20% (2 to 46) | 0.63 |
| 2016 | remained 0% excise | Bottled water, untaxed | 27.27 | 11.24 | 16.03 (11.06 to 21.05) | 143% (69 to 334) | 4.51 |

Note: *cross price elasticity. Results are adjusted for visitor numbers, GDP per capita and exchange rate T$/US$. p = person

### Sensitivity tests

#### Import sensitivity analyses

Six pre-planned and four post-hoc sensitivity analyses were used to test the robustness of primary analysis. The effect of these model changes on the percentage change in sweetened beverage import volumes after the tax change was assessed. Sensitivity tests were:

1. Three month lag in effect: There was a change that import volumes took more than three months before they responded to the tax changes for example reflecting time to finish current stock, place new orders and transport time. A three month lag time in import volumes impacts was modelled by reducing the follow-up after 2016 and 2017 taxes to nine months. The time lag period was ignored in the calculation of pre-existing trends.
2. Maximum lead-in period of three years: The lead-in period was reduced to three years maximum, according to a pre-planned analysis. This was because time points further away from the tax change may be more susceptible to external factors (time varying confounding) [3].
3. The proportion of sweetened beverages no longer subject to the excise after the 2017 tax change were estimated and subtracted from sweetened beverage import volumes.
   In 2017 the excise on sweetened beverages with ≤5g/100ml of sugar was replaced with a 15% tariff. The value of the sweetened beverage imports that were charged a 15% tariff was used to estimate the proportion of artificially sweetened beverages. However, this assumes that there were no imports of low-sugar beverages before the 2017 tax.
4. Major cyclones may have impacted demand and transport of beverage imports, particularly in the immediate aftermath. Therefore adjustment was made for each of Cyclone Gita (February 2018, Tongatapu) and Cyclone Ian (January 2014, Ha’apai). This was done by introducing a dummy variable to the model, which signalled the month following the cyclone.
5. A model was run with no adjustment for autocorrelation to test for its impact on the model.
6. A model was run with no adjustment for confounding to test the effect of potential confounders in the model.
7. Quadratic for time: Time squared was added to the model, allowing the trend in import volumes to change exponentially. This contributed to a significantly better model fit in the sweetened beverage import analysis.
8. Population: The population numbers in the data were fixed to an average population of 103,000 for all months. This was done to assess the influence of population change in the model, because there was a concern that the 2016 population was less than predicted by 2011 projections that were used in this study.
9. Quarterly analysis: All study outcomes and covariates were modelled with quarterly data, to reduce the monthly variation and assess the impact on study power.
10. The effect of the very steep trend change post 2016 tax was removed from the model. This trend is likely to have been affected by the very low import volumes immediately after the 2016 tax was introduced.

Table G summarises the sensitivity test results for the impact of the tax increases on sweetened beverage import volumes in the first year. The majority of sensitivity tests gave similar results to the primary analysis. Of note, removing the post 2016 trend change from the model reduced the effect of the 2017 tax change by more than half from -62.5% (-73.1 to -43.4) to -25.9% (-47.4 to 14.4). The effect of the 2013 tax introduction was doubled when the lead in period was limited to three years from -10.4% (-23.6 to 9.0) to -21.0% (-31.4 to -5.9). There was no increase in the effect of the tax with a three month lag in effect.

**Table G: Sensitivity tests of the analysis comparing the percentage change in sweetened beverage import volumes in the first year of follow-up after the 2013, 2016 and 2017 tax changes in Tonga**

|  |  | 2013 | 2016 | 2017 |
| --- | --- | --- | --- | --- |
|  |  | % change (95% CI) | % change (95% CI) | % change (95% CI) |
| Primary analysis | Primary analysis, pre-post tax comparison, monthly | -10.4% (-23.6 to 9.0) | -30.3% (-38.8 to -20.5) | -62.5% (-73.1 to -43.4) |
| Sensitivity tests (as planned) | Three month lag in effect, with 9 months follow-up for 2016 & 17 taxes | **-3.1%~** (-26.9 to 50.0)~ | **-18.0%~** (-33.2 to 1.7) | -53.1% (-74.7 to 65.1) |
|  | Maximum lead in period of three years | **-21.0%*** (-31.4 to -5.9) | -37.4% (-44.4 to -29.1) | -68.7% (-75.6 to -57.9) |
|  | Estimate impact of removing ASBs after 2013 tax | -10.4% (-23.5 to 9.1) | -30.4% (-38.9 to -20.6) | -66.0% (-75.8 to -48.4) |
|  | Adjustment for Cyclone Gita (February 2018, Tongatapu) | -10.5% (-23.5 to 9.1) | -30.0% (-38.6 to -20.2) | -63.5% (-74.0 to -44.3) |
|  | Adjustment for Cyclone Ian (January 2014, Ha’apai) | -10.4% (-23.1 to 8.4) | -31.3% (-40.6 to -20.1) | -62.8% (-73.4 to -43.7) |
|  | Without adjusting for autocorrelation | -8.5% (-44.2 to 89.7) | -25.2% (-53.2 to 29.5) | -53.3% (-72.3 to -1.2) |
|  | Without adjusting for confounding | **-7.0%~** (-13.5 to 0.0) | -33.8% (-44.3 to -21.6) | -59.0% (-73.8 to -25.7) |
| Further tests (post hoc) | Quadratic term; time squared added as a covariate to the model (because there was a significantly better fit) | -13.2% (-30.3 to 17.8) | -34.4% (-52.0 to -2.3) | -63.2% (-74.0 to -43.0) |
|  | Remove effect of population change, fixed population of 103,000 | -11.0% (-23.7 to 7.8) | -31.0% (-39.4 to -21.4) | -62.7% (-73.2 to -43.9) |
|  | Quarterly analysis with quarterly outcome and covariate data | **-21.3%*** (-39.2 to 15.9) | -31.1% (-43.6 to -15.0) | -57.6% (-72.5 to -23.5) |
|  | Remove the post 2016 tax trend | **3.7%~** (-24.2 to 58.5) | -27.9% (-44.7 to -4.4) | **-25.9%~** (-47.4 to 14.4) |

Notes: adding a covariate for the number of young adults in the population was also tested as a sensitivity test however this model could not be run due to convergence. Larger declines (eg a third or more different) are in bold and coloured **marked** with an astrix (*) while smaller declines (eg a third or more different) are also **marked** (~).

#### An alternative method for measuring tax size

Tax changes were also calculated from revenue collection to compare with tax changes calculated from import unit values as a sensitivity test. The size of each volumetric tax was converted to a percentage using revenue data from Tonga Customs. The sum of tariff and excise revenue as a proportion of the import value was calculated for each beverage import category, thus averaging the different tax rates in beverage sub-categories (eg, sweetened drinks with different concentrations of sugar). The added advantage of this method was the ability to calculate the size of the taxes averaged across the total import category irrespective of differences in sugar concentration within the category.

Using this alternative method, tax increases were approximately 20% smaller, possibly because of tax exemptions for some beverages for example those with ≤5g sugar/100ml in 2017 (Table H). If these tax changes were used in the main analysis, we would expect elasticities would be slightly greater.

**Table H: SSB tax changes calculated from changes in revenue collection one year pre- and post-tax change**

|  | **Tax changes** | **Beverage category** | **Size of the previous year’s tax as a percentage of import value** | **Size of previous year’s tax per import volume (P$/L)** | **Size of the new tax as a percentage of import value** | **Size of new tax per import volume (P$/L)** | **Size of the tax increase (% point change)** |
| --- | --- | --- | --- | --- | --- | --- | --- |
| **2013** | 15% tariff → T$0.50/L excise | Sweetened beverages, including flavoured milk  HS 22.02 | 16% | $0.19/L | 39% | $0.50/L | 23% |
| **2016** | excise T$0.50/L →$1.00/L | Sweetened beverages, including flavoured milk  HS 22.02 | 35% | $0.50/L | 60% | $0.92/L | 25% |
| **2017** | excise T$1.00/L →$1.50/L if sugar >5g & ≤20g/100ml | Sweetened beverages, all sugar concentrations  HS 22.02 | 60% | $0.92/L | 82% | $1.41/L | 22% |
|  | 15% tariff → $1.50/L excise if sugar >5g & ≤20g/100ml | Juice, all sugar concentrations  HS 20.09 | 15% | $0.39/L | 55% | $1.33/L | 40% |
|  | 15% tariff → $4/kg excise | Powdered drink sachets  HS 1701.91.10 | 15% | $2.44/kg | 26% | $3.98/kg | 11% |

Notes: Calculated from annual tariff and excise revenue intake, import value and import volume. Values are averaged across all entries for each beverage import category in a one year period. Source: Customs revenue

### Modelling the counterfactual

In the 2013 to 2016 time period in Fig. 3 for sweetened beverage imports the counterfactual unexpectedly declined. However, when all three confounders were removed from the model, this was no longer the case (Table G). GDP per capita had a negative coefficient in the model (-0.002715), and it rapidly increased during the 2013 to 2016 time period. This somewhat reduced the effect associated with the 2013 tax change particularly in the second year, also affecting price results.

The model also predicted two very low measures of sweetened beverage volumes in the months after the 2016 tax change, as per measured import volumes at this time. These values were influential and caused an increase in the slope of observed sweetened beverage import volumes in the 2016 to 2017 time period, and an increase in the slope of the 2017 to 2018 counterfactual. This effect increased the estimated decline after the 2017 tax change. The impact of the 2016 trend changes was tested by removing it from the model in a sensitivity test, halving the 2017 taxes impact on sweetened beverage import volumes (Table G).

## Litter survey

### Methods

Beverage container litter surveys were done to examine the impact of beverages on the environment and collect indicative information on the types of beverages consumed in Tonga and their origin including the market share of imported compared to locally produced beverages. Retail surveys could have been used however litter was considered a better indicator of what was actually purchased and consumed. Litter surveys were carried out at three beaches and three streets in Tongatapu in October 2018 by the lead author (AT) and co-author VP. Locations were selected by prioritising frequently used areas namely accessible beaches, beach resorts, a rural village street, and two streets in Nuku’alofa, the main township (Figure B).

Information was collected on each survey location (via GPS recording) and for each type of litter (eg, via photographs) using study protocols. The development of the protocol for the beach litter survey was informed by a study of marine plastic bottle pollution [5] and the UN Environmental Program guidelines for marine litter surveys. [6] The protocol for the street litter survey was designed to be consistent with the beach survey protocol and also draws on the NZ litter survey methodology. [7, 8]

#### Beach litter survey protocol

1. The key outcome measure was beverage container density per kilometre of beach length.
2. Three beaches were selected based on a range of different uses and proximity to urban centres and tourist hotspots. Beaches with coral reefs were included, but beaches adjacent to islands or seawalls were excluded. [6]
3. Each survey location (transect) was expected to be about 200-500 m in distance. In some cases the distance was extended because very few beverage containers were found. The aim was to collect at least 10 containers per beach. Each transect was expected to cover 2,000m^2^ or more of beach (eg, 200m length x 10m beach width).
4. Equipment for the survey included a rubbish bag/sack, gloves, smartphone, GPS tracker and sturdy footwear.
5. A GPS tracker was used to record GPS coordinates to map out the route of the litter survey and measure the distance that was surveyed. The tracker was turned on at the start of the survey and switched off at completion. For back-up; photographs (tagged with date and location) were taken at the start and end of the survey, including any landmarks so that these points could be located on Google maps/ satellite pictures. Photographs were also useful for measuring the distance and recording start and end times.
6. Information on the environment was also recorded. Photographs were taken of the beach at the start of the survey to document features such as the beach width, vegetation, and driftwood density (which could trap bottles; and also obscure them). Any anti-litter signs or rubbish bins were photographed. Also any large collections of litter in one place were specifically photographed.
7. The researcher walked in a straight line along the high tide mark on the beach front, and then returned by walking along the same section of beach in the middle of the fore-dune area higher up the beach, for example along the edge of the trees and vegetation. The researcher searched a 5m width area comfortably with each pass, to identify any potential beverage container, but up to 10m width as necessary. In most tropical beaches this search covered the whole beach face and fore-dune area because the beach front was narrow.
8. Any drink containers (or cups, or fragments) observed larger than 2.5cm in maximal diameter were collected (because this size is considered visible litter). If it was not identifiable as a drink container, or it was not visible then it was not included. All the litter together, and each individual piece of litter was photographed in detail at the end of the search. The photograph recorded all imagery and written material and embossing visible on each container, and any marine growth. Litter was disposed of appropriately in rubbish receptacles after data collection was complete. There were some beverage containers that could not be collected (eg, if they were stuck under vegetation or rocks) and these were photographed in situ. If a container was sited within 3m of a rubbish bin, this information was recorded by photographing the container in situ in relation to the rubbish bin.

#### Street litter survey protocol

The street litter survey was carried out in the exact same manner, albeit with the following minor differences:

1. The primary outcome for this survey was the number of beverage litter items per kilometre of road, or per 1000m^2^. The latter measure is comparable to a litter survey that included New Zealand beverage packaging (<https://www.wasteminz.org.nz/wp-content/uploads/WasteMINZ-2015-National-Litter-Survey.pdf>).
2. Approximately three street survey locations were selected based on their proximity to frequently used areas such as urban centres and tourist spots. For example, main roads were a focus given their greater use. Each survey area was expected to be about 400 to 800m long (eg, 400m x 6m width = 2400m^2^). Some streets were known to be cleaned, typically in urban centres which may have reduced the amount of collected litter.
3. The GPS tracker was used to identify GPS coordinates and map the route. Photographs were taken of the street at the start and the end of the survey to measure distance, date and time.
4. Further photographs were taken to document the width of the kerbside, land use type (residential, urban, rural), location of shops and markets, other types of street use, and any anti-litter signs or rubbish bins.
5. The researcher walked down both sides of the street or road that was being surveyed. The area from the kerbing channel to the property boundary was searched to identify any potential beverage containers or fragments (larger than 2.5 cm was considered visible litter).
6. All eligible beverage containers were collected and photographed in detail at the end of the survey as described for the beach litter survey.

VP assisted AT in all three beach surveys and one of the street litter surveys. For the street litter surveys, one person examined each side of the road. In beach surveys one person examined the fore-dune area of the beach and the other walked the high tide line. Occasionally additional rubbish was collected on the return walk. Two of the Tongan beaches were wide, and our survey strategy was not expected to identify all of the existing beach litter on these beaches.

#### Analysis

Information from both the beach and street litter surveys was extracted and recorded in standardised Excel spreadsheets. The location spreadsheet was used to record location and sampling information including; location name, time and date, length and width of beach searched, remoteness and accessibility, nearest rivers, nearest ports and urban centres, and any litter signage or rubbish bins. The litter spreadsheet was used to record packaging (UNEP code, UNEP description, description), product type (beverage, volume, alcoholic or not, beverage category, brand), manufacturer, country of origin, marine/other growth (eg, algae or bryozoans for beach litter) and whether litter was collected from within 3m of a rubbish bin (because it may have fallen out). This was done using information on the labels or other identifiable features on the bottle or lid of the container (text, embossing, and symbols) or comparison of container features with other containers collected from the same location.

Litter was classified according to the UNEP [6] litter classifications with the addition of whether the beverage was alcoholic or not. Table J outlines how the UNEP classifications correspond to New Zealand (NZ) litter survey classifications [7, 8] for beverage containers and their packaging.

Litter density was described by location and beverage type. Country of origin was described by beverage type, where this information was available on the beverage label or by inferring this information from equivalent beverage containers also collected from the same island.

**Table I: Litter classifications used in the analysis**

| **Marine litter classifications [6], litter >2.5cm in diameter on any dimension** | | | **NZ urban litter classifications [8], visible (>6cm^2^) and bulky litter (bigger that can fit in a standard rubbish bin)** | | |
| --- | --- | --- | --- | --- | --- |
| **Material** | **Code** | **Description** | **Primary category** | **Secondary category** | **Description** |
| Plastic | PL02 | Bottles < 2 L | Drink container (non-alcoholic) | PET | Clear #1 plastic drink bottles, such as those used for soft drinks and bottled water |
|  |  |  | Drink container (non-alcoholic) | HDPE | #2 plastic drink containers, such as those used for milk products |
|  |  |  | Drink container (alcoholic) | PET | #1 plastic alcohol bottles, including some beer, wine, spirits, and cider bottles |
| Metal | ME03 | Aluminium drink cans | Drink container (non-alcoholic) | Aluminium Cans | Soft drink and juice cans |
|  |  |  | Drink container (alcoholic) | Aluminium Cans | Primarily beer or ready-to-drink (RTD) cans |
| Glass & ceramic | GC02 | Bottles & jars [only include beverage containers] | Drink container (non-alcoholic) | Glass | Glass bottles such as used for soft drinks and juices |
|  |  |  | Drink container (alcoholic) | Glass | Beer, wine, spirit bottles etc |
|  |  |  | Drink container (alcoholic) | Multi-material/ other | Any alcohol container made of another material (such as ceramics) or more than one material |
| Plastic | PL06 | Food containers (fast food, cups, lunch boxes & similar) [only include beverage containers] | Disposable cup (hot and cold) | None | Disposable cups made of any material, including paper, expanded polystyrene, and plastic, such as coffee cups, straws, cup lids, milkshake cups, and soft drinks |
| Paper & cardboard | PC03 | Cups [for beverages], drink containers [only beverage containers, exclude food trays, food wrappers, cigarette packs] |  |  |  |
|  |  |  | Drink container (non-alcoholic) | Aseptic and gable-top | Tetra Pak-type packaging boxes for drinks that do not require refrigeration, such as juices, and coated cardboard cartons for drinks that do require refrigeration, such as milk |

**Figure B: Litter survey locations in Tongatapu (most populated island in Tonga), using Google Earth**


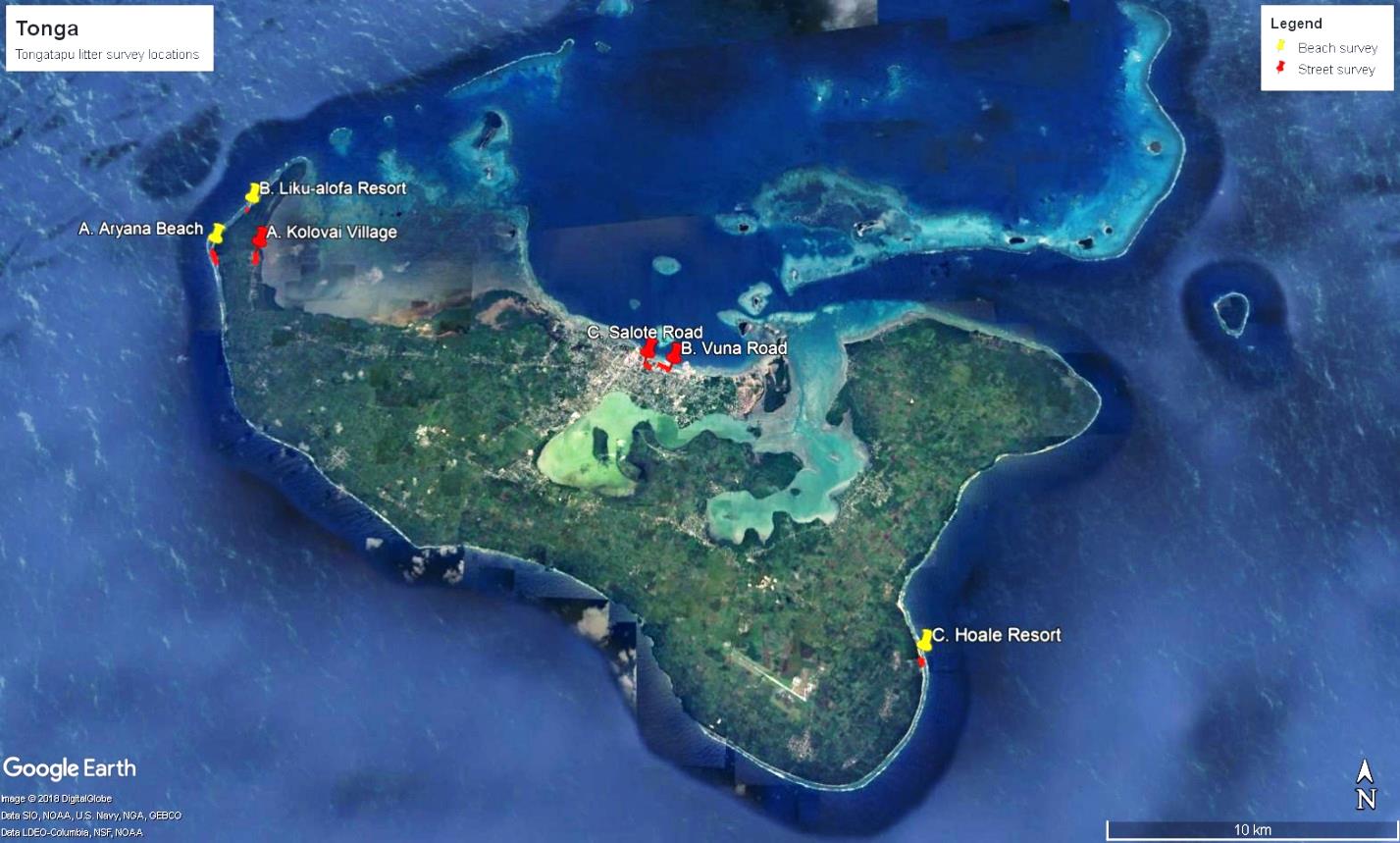


### Results of litter distribution and origin

In total, 455 drink containers and associated fragments were collected from 1031m of beach, and 87 drink containers and fragments were collected from both sides of 1242m of road. The density of litter on the beaches was very high with an average of 592 items/km, but much less on the roads with an average density of 71 items/km. A large proportion of roadside litter was from SSBs (64%), whereas beach litter was commonly from SSBs (34%) and alcohol (33%). Beaches B and C were adjacent to a tourist resort, and Beach A was adjacent to a closed resort. Roads B and C were in Nuku’alofa and at the time of the study were being frequently cleaned of litter by street cleaners.

The country of origin of beverage manufacture was identified for 477/542 (88%) of the containers/ fragments. Among these items, 18% were manufactured in Tonga and 82% from outside of Tonga. Beverages manufactured in Tonga corresponded to half of the water bottles (50%, total n=118), one in eight alcohol containers (12%, n=142), one in ten juice containers (10%, n=10) and one in twenty SSB containers (5%, n=203). Of all beverage litter, 17% originated from other PICTs (eg, Fiji), corresponding to 34% of SSBs and 22% of juice. Litter from milk (n=3) and ASBs (n=1) was rarely collected and was all imported. All litter collected was disposed of appropriately, with the support of local residents.

### Market share of locally produced soft drinks

Manufacturing data can also be used to estimate market share and compare with the litter survey findings. The value of soft drink manufacturing in quarter two 2017, as a ratio to import value (CIF) of sweetened drinks in the same quarter, was T$39,188 / T$1,673,000 = 2.3%. Considering both findings the approximate market share of locally produced soft drinks towards the end of the study period in mid-2018 was likely to be about 5% or less.

## References

1. Teng AM, Jones AC, Mizdrak A, Signal L, Genç M, Wilson N: **Impact of sugar-sweetened beverage taxes on purchases and dietary intake: Systematic review and meta-analysis.** *Obes Rev* 2019, **20:**1187-1204.

2. Stawowy W: **Calculation of ad valorem equivalents of non-ad valorem tariffs - methodology notes.** pp. 1-11: Division on International Trade in Goods and Services, and Commodities UNCTAD 2001:1-11.

3. Lopez Bernal J, Cummins S, Gasparrini A: **Interrupted time series regression for the evaluation of public health interventions: a tutorial.** *Int J Epidemiol* 2017, **46:**348-355.

4. Penfold RB, Zhang F: **Use of interrupted time series analysis in evaluating health care quality improvements.** *Acad Pediatr* 2013, **13:**S38-44.

5. Smith SDA, Banister K, Fraser N, Edgar RJ: **Tracing the source of marine debris on the beaches of northern New South Wales, Australia: The Bottles on Beaches program.** *Mar Pollut Bull* 2018, **126:**304-307.

6. Cheshire A, Adler E, Barbière J, Cohen Y, Evans S, Jarayabhand S, Jeftic L, Jung R-T, Kinsey S, Kusui ET, et al: **Guidelines on Survey and Monitoring of Marine Litter Regional Seas Reports and Studies No. 186 IOC Technical Series No. 83.** In *UNEP Regional Seas Reports and Studies, No 186; IOC Technical Series No 83: xii + 120 pp* (United Nations Environment Programme / Intergovernmental Oceanographic Commission ed. pp. 1-117. Nairobi; 2009:1-117.

7. **The Packaging Forum’s National Litter Survey 2014-15** [<https://www.wasteminz.org.nz/wp-content/uploads/WasteMINZ-2015-National-Litter-Survey.pdf>]

8. Waste Not Consulting: **National Litter Survey 2014-2015: Summary of Results.** (Waste Not Consulting Ltd ed. pp. 1-12; 2015:1-12.
