# Supplementary material for: Sweetened beverage taxes and changes in beverage price, imports and manufacturing: interrupted time series analysis in a middle-income country
Source: Int J Behav Nutr Phys Act. 2020 Jul 9;17:90. doi: 10.1186/s12966-020-00980-1 (PMC7350205; doi:10.1186/s12966-020-00980-1)
Supplement: Supplementary file 2 — Additional file 2. Study protocol, also available online at http://hdl.handle.net/10523/9432. [file 12966_2020_980_MOESM2_ESM.pdf]

Impact of sugar-sweetened beverage tariffs and excise on taxed and untaxed beverage trade volumes to the Cook Islands and Tonga: Pre-analysis study protocol

The structure of this protocol is adapted from the SPIRIT 2013 Checklist of Recommended items to address in a clinical trial protocol, wherever relevant to this interrupted time series study design.

Administrative information

Title	1	Impact of sugar-sweetened beverage tariffs and excise on taxed and untaxed beverage trade volumes to the Cook Islands and Tonga: Interrupted times series analysis
Protocol version	3	Version 1.0, 11 March 2019
Funding	4	Health Research Council of New Zealand: Clinical Research Training Fellowship<br>Otago Global Health Institute, University of Otago for travel funding<br>Division of Health Sciences, University of Otago for travel funding
Roles and responsibilities	5a	First author is Andrea Teng, Senior Research Fellow, PhD Candidate, University of Otago, Wellington
	5b	Study is supported by the Secretariat of the Pacific Community, Dr Paula Vivili
	5c	The study funders and sponsors had no role in the study design; collection, management, analysis, and interpretation of data; writing of the report; and the decision to submit the report for publication
	5d	Other team members and roles:<br>Prof Nick Wilson, primary supervisor<br>Prof Louise Signal, supervisor<br>Dr Murat Genç, supervisor<br>Dr Viliami Puloka, advisor<br>Ms Karen Tairea, Cook Islands

Introduction

Background and rationale	6a	This study will contribute to the international literature of policy evaluation studies that examine the impact of sugar-sweetened beverage (SSB) taxes on sales, purchasing and dietary consumption (summarised in Chapter 4 systematic review meta-analysis, under review). Further research is needed to understand whether SSB taxes are associated with changes in consumption of untaxed beverages such as water and milk, and how this varies between contexts. This analysis will examine the association between SSB taxes and substitution in trade to untaxed beverages.<br><br>Furthermore, the study will contribute to the low number SSB tax evaluations from Middle Income countries and Small Island Developing States. Tonga and Cook Islands are Upper Middle Income countries, however the Cook Islands is likely to graduate from Upper Middle Income to High Income status in the future, after a delay was granted until the end of 2018 (due to a lack of GNI data).<br><br>The aim of the study was to examine the impact of SSB import tariff and excise changes in the Cook Islands and Tonga on:<br>1. shelf price of selected taxed and untaxed beverages,<br>2. import volumes of taxed and untaxed beverages,<br>3. sales of taxed and untaxed beverages (in Cook Islands where data were available), and<br>4. manufacturing levels of taxed and untaxed beverages (in Tonga where data were available)
	6b	The primary comparison will be pre–post taxation, however secondary analyses will examine SSB tax impacts compared to trends in untaxed control food groups (eg, sugary foods), and/or control jurisdictions where there were no SSB tax changes during the same time period.
Objectives	7	Based on economic theory, it is expected that tax changes of >10% will increase the shelf price of taxed beverages more than existing trends; and subsequently that the importation and sales volumes of taxed beverages will decline compared to previous trends. In a similar way it is expected that the importation and sales volumes of untaxed beverages would increase in response to SSB tax changes. Local manufacturing of taxed beverages may increase if they are sold at a cheaper rate than importer beverages.
Study design	8	Interrupted time series analysis[1,2] with and without controls.

Methods: Participants, interventions, and outcomes

Study setting	9	Cook Islands and Tonga were selected as jurisdictions of interest. Further information on these settings is described in earlier thesis chapters (written pre-analysis).<br><br>The World Bank was commissioned by the Tonga Government to evaluate health taxes on food and beverages. The World Bank evaluation work was underway in 2018, in a similar timeframe to this study. The author approached the World Bank to explore if there would be any overlap or areas for collaboration. The World Bank recommended that the analysis outlined here be carried out independently to their work. Results and methods from the World Bank SSB tax evaluation were not available to the author at the time of protocol publication.
Eligibility criteria	10	All Pacific Island Countries and Territories (PICTs, ie all 22 Pacific members of the Pacific Community) were eligible for selection in this study. Eight of these jurisdictions have introduced excise taxes on SSBs since 2000.<br><br>Cook Islands and Tonga were selected because in both countries there have been recent increases in SSB taxes to levels greater than 10 percent, and there were data available on import trends and from household expenditure surveys. Both countries have been largely dependent on trade for SSB consumption, at least historically, improving the validity of trade as an estimate of SSB consumption.
Interventions	11a	We were particularly interested in SSB tax changes that were greater than 10 percent as these are likely to have more impact on measurable price changes and health outcomes. However, all the major SSB tax changes in each jurisdiction were studied, at least partially, with the expectation of minimal or zero trade declines when tax changes were small or negative.<br><br>For example, the 2014 Cook Island SSB excise aimed to be revenue neutral, and had an average negative effect on the sweetened-beverage tax, as calculated from import values and soft drink sugar consumption. Furthermore, the decline in the level of tax with the excise was even greater for zero sugar beverages which were not included in the excise. The impact of the excise was examined on sales data that could distinguish between regular and low sugar beverages. In Tonga the transition from a 15% import tariff to a T$0.50/L excise appeared to have a small impact (from 15% to 24% ad valorem equivalents [AVE]), thus a relatively small change in SSB price, and levels of trade or manufacturing was expected.

A significant increase in manufacturing of SSBs or untaxed beverages for the local market would have important implications for the population consumption of SSBs. For this reason information was sought from stakeholder interviews, existing reports, price surveys and litter surveys of beverage containers to glean information on the 2018 levels of local manufacturing. Where local manufacturing levels of a specific beverage were estimated to be greater than 10% of the market at the time of the SSB tax change, then the time series analysis of import trends will not be carried out.

11d Concomitant interventions, policy changes or global trends will not necessarily exclude the analysis of SSB tax changes, but these interventions will be described in detail and discussed for their impact on results.

Outcomes	12	Outcomes at one year were compared with the counterfactual outcome level which was based on pre-existing trends. This is because the impact of the tax is expected to have occurred by this point, and trends have only been exposed to influence from other external factors for a limited time period. Even a small change in consumption at the population level could potentially lead to health benefits over time. However, substitution effects may affect the health benefits and thus substitution was also studied here. The outcomes were:

Cook Islands

- Exports from NZ to Cook Islands (litres per residential population), with weighting of NZ Exports to the Cook Islands by the inverse of the proportion of the worldwide exports to Cook Islands that were from NZ for that product
  - SSBs total (includes diet in 2008 and 2012)
  - Untaxed beverage total
  - Untaxed beverage subcategories; water, milk, juice, powdered drink sachets
- Price changes, two Coca-Cola beverages (NZ$ per item)
- Sales data of sugar-sweetened and low calorie soft drinks and energy drinks, pre-post 2014 tax change (volumes sold)

Tonga

- Imports into Tonga (litres per population)
  - SSBs total L/population (excludes zero sugar in 2017 tax)
  - SSB subcategories; sweetened drinks, drink sachets and juice (2017 only)
  - Untaxed beverages total
  - Untaxed beverages subcategories; water, milk, sachets & juice (2013, 2016 only), diet drinks (2017 only)
- Price changes, selected taxed and taxed beverages (T$ per item)
- Manufacturing levels of soft drinks and of water (T$) per quarter (2017 only)

The primary outcome for trade analyses was the change in the combined number of litres imported per population (or sales volumes, or manufacturing value) in the first year after the tax change, calculated using the changes in the level and the trend comparing modelled data to the counterfactual which is based on pre-existing trends, for each quarter in that year. For price outcomes, the average price change in the first four quarters post-tax was the primary outcome. Both the absolute change (rate difference [RD], measured in litres per person, or dollars) and the relative change will be calculated (rate ratio [RR] or percentage change). Confidence intervals for these measures will be calculated using Monte Carlo simulation, using the coefficients and their standard errors from the generalised least squares model, and allowing for the correlation between correlates.

The RR effects will be used to calculate tax elasticities, that is the percentage change in consumption post-tax compared to the counterfactual, divided by the size of the SSB tax change as a percentage. The percentage sizes of import tariff changes (eg, ad valorem) were readily available. Excise taxes changes (ie, volumetric taxes, applied per litre of sweetened beverage) will be converted into estimated ad valorem equivalents (AVEs) using trade unit values (local currency unit per litre) in the year of tax introduction, as has been done elsewhere for calculating AVEs from trade databases.[3] There is the risk that these elasticities will overestimate the AVE and underestimate tax elasticities, however it was the only method available and comparable to the method used in the meta-analysis associated with this study. Locally sold Coca-Cola will be used as the reference soft drink to identify soft drink sugar concentration and identify the category of SSB taxes that applies to sweetened drinks.

Where it is possible, outcomes will also be considered at two years post tax changes using the same methods described above.

A set of pre-specified sensitivity tests will be used to assess the robustness of the key SSB trade results for Cook Islands and Tonga.

1. Price and trade analyses using a monthly time period where possible, without adjustment for any missing potential confounding variables that were not available monthly.
2. Allow for the impact of major cyclones to see if any significant changes eg, Cook Islands in 2005 and Tonga in 2018. If this makes a significant impact then this additional variable will be included in the final model.
3. Allow for a three month lag time in the changes to import data, reflecting time it might take to run out of stock, place new orders and for these to arrive. Personal communication with key informants was that the effect of SSB tax on price was noticed within three months, so the default analysis will not include any time lag.
4. Test the robustness of the NZ export weighting, by limiting the study to NZ export volumes alone (rather than weighting NZ export volumes up to make them represent worldwide exports to Cook Islands). The accuracy of the UNComtrade data from where the weights are sourced is questionable eg, due to missing data. So this will test to what degree the weights affect key study findings.

5. Slightly more limited pre-tax and post-tax time periods may be selected (eg, a maximum of three years to retain adequate power) to investigate the impact of removing time points a long time away from the tax change, which may be more susceptible to external non-SSB tax factors. The default analysis includes all available data to maximise study power. This is particularly import if historical trends have changed over time.[1]
6. Examine the impact of removing autocorrelation from the model.

Timelines	13	The timeframes for the pre-post comparisons are summarised in the figure below. Follow-up may be extended if new data becomes available for the required variables. Follow-up timeframes in analyses which include a control comparison may vary due to the available comparable data.
Sample size	14	The ideal would be to have had 12 time-points[4] pre-tax for which to compare with post-tax trends over 12 time-points, however this was not always possible given the available data and timing of tax interventions. If there were more than 12 time-points pre-tax all time-points back to 2000 were included and if there were less the analysis was still carried out, with the acknowledgements that it has less power of which to determine a statistically significant change (unless the data were very consistent from quarter to quarter such as the average shelf price information based on several stores).

Quarterly data was used wherever possible for several reasons

- To limit the variability (random noise) between time points in relatively small jurisdictions, while also maximising study power. Annual data reduces the number of time points available and the statistical power.
- Data on several model variables were only available quarterly and not more frequently
- To allow assessment of seasonal variability (as opposed to annual data)

Figure: **Schedule of pre-tax and post-tax follow-up and reporting**

INTERVENTION TIMEPOINT	*Size of the tax change*	*2000*	*2001*	*2002*	*2003*	*2004*	*2005*	*2006*	*2007*	*2008*	*2009*	*2010*	*2011*	*2012*	*2013*	*2014*	*2015*	*2016*	*2017*	*2018*	*2019*
		Study period																			
TRADE & PRICE ANALYSES *(quarterly data)*																					
**Cook Islands 2008** tariff 40% →60%	20% import price									X											
**Cook Islands 2012** tariff 60% →75%	15% import price													X							
**Cook Islands 2014** Tariff 77% → NZ$9.37/kg of sugar, excise	-18% AVE															X					
**Tonga 2013** 15% tariff → T$0.50/L excise	9% AVE														X						
**Tonga 2016** excise T$0.50/L →$1.00/L	47% AVE																	X			
**Tonga 2017** excise T$1.00/L →$1.50/L	59% AVE																		X		
SALES ANALYSES *(annual data only)*																					
**Cook Islands 2014** Tariff 77% → NZ$9.37/kg of sugar, excise	-18% & -77% AVE (zero sugar)															X					
MANUFACTURING ANALYSES *(quarterly data)*																					
**Tonga 2016** excise T$0.50/L →$1.00/L	47% AVE																	X			

		Study period																			
**INTERVENTION TIMEPOINT**	***Size of the tax change***	***2000***	***2001***	***2002***	***2003***	***2004***	***2005***	***2006***	***2007***	***2008***	***2009***	***2010***	***2011***	***2012***	***2013***	***2014***	***2015***	***2016***	***2017***	***2018***	***2019***
**Tonga 2017** excise T$1.00/L →$1.50/L	59% AVE																		X		

Notes: ‘X’ marks the timing of the tax change, usually mid-year. Black lines indicate the timeframe of trade/sales/manufacturing analyses. Yellow lines indicate the timeframe of the price analyses. Red lines indicate potential trade analyses if data can be updated.

AVE is calculated using the import value per litre, and then applying the specific tax to sweetened drinks. The concentration of sugar used to calculate the Cook Islands excise was estimated from a standard concentration of Coca-Cola soft drink.

Methods: Assignment of interventions

Confounding:

Control selection	16a	The primary analyses will not include control comparisons, however two types of control will be used to test robustness of study findings.<br><br>An ideal control would be a neighbouring jurisdiction with very similar socio-economic/cultural context and all the required available data, where no SSB tax was introduced during the study time period. In some cases it may be possible that Cook Islands and Tonga can each act as a control to the other jurisdiction. This is useful because the context, SSB tax, trade and potential confounding datasets for both countries will be available. This approach will be explored for example for the 2012 tariff increase in Cook Islands and for the 2016 excise increase in Tonga. This control comparison was not considered for the main analyses because the follow-up periods are likely to be more limited, and a control was not available for all tax change interventions.<br><br>Furthermore, within-country controls will also be used by selecting an untaxed trade category from the same country's trade datasets. Untaxed beverages are not a suitable control because they might respond to the SSB tax changes as substitutes and trade volumes of these beverages may increase. Instead, other sugary snack food items will be selected, at least partially because of their discretionary and sweet nature. The trade categories will comprise icecream, chocolate, sweets, crackers/biscuits and cake; but icecream was excluded from this group when the excise tax in Tonga was also applied to icecream (2016). There is limited evidence of any relationship between SSB taxes and substitution effects to sugary snack foods.[5,6] But if there is an affect it appears to be quite small eg, sweets/desserts/chocolate/chewing gum (cross price elasticity 0.03, $p<0.001$)[7], and cookies/other snacks (cross price elasticity 0.01, $p<0.001$).[7] However, because of the risk that sugary snacks could be a substitute, the default analyses will not include the sugary snack control.
Confounder adjustment	16b	Confounding will be adjusted for by including quarterly information on these factors in the model for trade, sales and price analyses;<br>- International visitor numbers<br>- GDP (in local currency units)<br>- Season (each quarter as a separate category)<br>- (Cyclone events causing substantial damage if any significant impacts are detected)

Data collection methods	18a	Monthly Cook Islands trade data (exports to Cook Islands from New Zealand) are freely available from the StatsNZ website (for all years in the study period; 2000 to present). The vast majority of imported products to the Cook Islands come from New Zealand, and this is particularly the case for the sweetened beverage category (UNComtrade).<br><br>Similar data was available from Tonga Customs. Monthly beverage import volumes data back to 2009 were provided, with the addition information on the different revenue streams for each beverage category.<br><br>Information was gathered on tax rates and import volumes for each beverage category, categorised by the international Harmonised System of coding used for analysing trade flows. The key beverages categories are summarised below, along with information on sugary snacks and alcohol categorisation. Sometimes categorisation varied between countries and so categories were validated with Customs officials who provided the information below. Favoured milk was included in sweetened drinks in Tonga, and in NZ trade data (personal communications with respective customs agencies).<br><br>2202 Sweetened drinks (includes regular and low calorie drinks)<br>2201 Water<br>2009 Juice<br>0401.00 to 0401.29 Milk (NZ, for Cook Islands)<br>0401.10 AND 0401.20 Milk (Tonga)<br>2106.90.9921K Powdered drink sachet (NZ)<br>1701.91.10 Powdered drink sachet (Tonga, personal communication Customs, 3/11/18)<br>2106.90.9979A Drink syrups, cordial concentrates and food preparations not elsewhere specified or included (NZ not specific to beverage preparations)<br><br>*Available for Cook Islands, but currently pending for Tonga:*<br>2106.90.00 Cordial concentrates (Tonga)<br>1704 Sweets and chewing gum<br>1806.20 to 1806.90 Chocolate<br>1905.20, 1905.30, 1905.31, 1905.32 Biscuits (includes crackers)<br>1905.90.0909 Cake (NZ)<br>2105 Icecream<br>2203 OR 2206 Beer/cider<br>2204 OR 2205 Wine<br>2208 Spirits/mixes

		Quarterly sales data (T$) were available for water and soft drinks from Tonga Statistics Department from 2015 to Q2 2017. Further updates will be requested for the next year of data. Some earlier years were also available but there was a large gap in data collection up to 2015.<br><br>Sales data for soft drinks and energy drinks, and whether they were regular sugar or low sugar products, were provided by Cook Islands Trading Company (CITC) from 2012 to Q2 2017.<br><br>Tonga and Cook Island Department of Statistics provided further quarterly data on population projections, price data of specific beverages included in the CPI calculations, GDP, visitor numbers and CPI, for differing time periods.
Data management	19	Data for each country and quarter will be saved into an Excel spreadsheet and imported into R for the analysis. The time series trends of each variable will be examined to check data quality and for any missing information or sudden changes. If there is any vital missing data, consideration will be given to extrapolation or imputation.

Statistical methods	20a	The statistical model of choice will be generalised least squares regression (GLS) for segmented regression analysis. This function fits a linear model and the errors are allowed to be correlated and/or have unequal variances. Testing and adjustment for autocorrelation and moving averages will be informed by the unadjusted model using the Durbin-Watson test, a graph of the residuals and a plot of the autocorrelation function and partial autocorrelation function.<br>GLS models with and without adjustment for autocorrelation will be considered. If the autocorrelation adjusted model is significantly different from the unadjusted model (log-likelihood ratio test), then the model with the best fit to the data will be selected (lowest AIC).<br><br>The model will be adjusted for potential confounding from GDP, visitors numbers and season (whether or not these factors have a significant impact on the model). Multiple intervention points in one country will be evaluated within the same model wherever possible.<br><br>Modelled trade outcomes and counterfactual results will be plotted by time pre- and post- intervention. These methods will be applied to all outcomes.<br><br>All analysis will be done using R.<br><br>Examples of models are presented in the appendix to this document.

	20b	Methods for sensitivity analyses and other additional analyses.<br><br>1. Will use a dataset with monthly trade or price data. Season will be coded in 12 months. Any confounding variable without monthly data will be excluded.<br>2. A dummy variable will be added to the model coded one for the quarter of major cyclone damage in the island nation of the study, and otherwise the variable will be coded zero.<br>3. The impact of the tax change will be coded to begin one quarter after the legislation change to allow for any potential short delays between law change, ordering, shipping time and warehouse storage time. Pre-existing trends will be calculated using the same data, thus the first quarter post-tax will drop out of the model and analysis.<br>4. A sensitivity analysis for the Cook Islands will be done with no weighting on the NZ export data. The quality of UN Comtrade data to produce the weighting variables is questionable and this change will test the dependence of the model on these weighting variables.<br>5. The evaluation will be limited to the three years pre-tax and up to three years post-tax. This is to reduce the effects of earlier and later outcomes that are more susceptible to other simultaneous trends and events.<br>6. Remove the autocorrelation adjustments from the model and compare study outcomes.

Ethics and dissemination

Research ethics approval	24	Ethics approval was obtained from the University of Otago, and from the Cook Island and Tonga Governments.
Protocol amendments	25	Any modification to this protocol will be reported in the final published article.
Declaration of interests	28	AT, NW, LS and MG declare no conflict of interests.
Access to data	29	The final analysis dataset will be made publically available, if agreement can be secured from the contributing agencies. No contractual agreements were made to access trade data used in these analyses.

Dissemination policy	31a	Preliminary results will be shared with key health and other stakeholders in Cook Islands and Tonga. This will involve the arrangement of visits to present study findings.<br><br>Results will be submitted to a peer-reviewed international journal. At the time of any publication a media release will summarise key findings to the public. Key New Zealand and Pacific stakeholders will be emailed the results and presentations will be made at national/regional/international meetings and conferences.
	31b	Standard authorship guidelines will apply. Local research collaborators will be encouraged to contribute to the manuscript development and to become co-authors. Acknowledgements will fully acknowledge the many contributors to this work.
	31c	The public will be able to access the full protocol on the University of Otago website. The plan is to grant public access to the full protocol, statistical code, and study datasets (when permissions for the later can be secured).

Notes

Amendments to the protocol should be tracked and dated. The SPIRIT checklist is copyrighted by the SPIRIT Group under the Creative Commons "Attribution-NonCommercial-NoDerivs 3.0 Unported" license.

References

1. Lopez Bernal J, Cummins S, Gasparrini A. Interrupted time series regression for the evaluation of public health interventions: a tutorial. *Int J Epidemiol* 2016.
2. Penfold RB, Zhang F. Use of interrupted time series analysis in evaluating health care quality improvements. *Acad Pediatr* 2013; **13**(6 Suppl): S38-44.
3. Stawowy W. Calculation of ad valorem equivalents of non-ad valorem tariffs - methodology notes: UNCTAD, 2001.
4. Wagner AK, S.B.Soumerai, Zhang F, Ross-Degnan D. Segmented regression analysis of interrupted time series studies in medication use research. *Journal of Clinical Pharmacy and Therapeutics* 2002; **27**: 299-309.
5. Finkelstein EA, Zhen C, Bilger M, Nonnemaker J, Farooqui AM, Todd JE. Implications of a sugar-sweetened beverage (SSB) tax when substitutions to non-beverage items are considered. *J Health Econ* 2013; **32**(1): 219-39.
6. Waterlander WE, Ni Mhurchu C, Steenhuis IH. Effects of a price increase on purchases of sugar sweetened beverages. Results from a randomized controlled trial. *Appetite* 2014; **78**: 32-9.
7. Guerrero-Lopez CM, Unar-Munguia M, Colchero MA. Price elasticity of the demand for soft drinks, other sugar-sweetened beverages and energy dense food in Chile. *BMC Public Health* 2017; **17**(1): 180.

Appendix: modelling examples

Primary analysis using GLS modelling

An example of the simplest interrupted time series segmented regression model with one intervention, and adjustment for confounding:

$$Trade(L/pop) = \beta_0 + \beta_1 . time_t + \beta_2 . tax_t + \beta_3 . tax\_trend_t + \beta_4 . GDP_t + \beta_5 . visitors_t + \beta_6 . season_t + \in_t \tag{1}$$

Where:
β_0 = intercept
β_1 = prexisting trend before the intervention
β_2 = level change after the first tax intervention
β_3 = trend change after the first tax intervention
β_4 = coefficient for the impact of GDP on trade
β_5 = coefficient for the impact of visitor numbers on trade
β_6 = coefficient for the impact of season on trade, categorical variable
$\in$ = error term

Example of a model with two interventions (primary analysis):

$$Trade(L/pop) = \beta_0 + \beta_1 . time_t + \beta_2 . tax_{1_t} + \beta_3 . tax_{trend1_t} + \beta_4 . tax2_t + \beta_5 . tax_{trend2_t} + \beta_6 . GDP_t + \beta_7 . visitors_t + \beta_8 . season_t + \in_t \tag{2}$$

Where;
β_0 = intercept
β_1 = prexisting trend before the intervention
β_2 = level change after the first tax intervention
β_3 = trend change after the first tax intervention
β_4 = level change after the second tax intervention
β_5 = trend change after the second tax intervention
β_6 = coefficient for the impact of GDP on trade
β_7 = coefficient for the impact of visitor numbers on trade
β_8 = coefficient for the impact of season on trade, categorical variable
$\in$ = error term

Example of a model where outcome is compared to a control group (secondary analysis):

$$Trade(L/pop) = \beta_0 + \beta_1 . time_t + \beta_2 . group_k + \beta_3 . group_k . time_t + \beta_4 . tax_t + \beta_5 . tax\_trend_{jt} + \beta_6 . tax_t . group_k + \beta_7 . tax\_trend_{jt} . group_k + \beta_8 . GDP_t + \beta_9 . visitors_t + \beta_{10} . season_t + \in_{ikt} \tag{3}$$

Where;
β_0 = intercept
β_1 = prexisting trend in the control group before the intervention
β_2 = level of trade at the start in the intervention group compared to the control
β_3 = group trend pre intervention relative to the control
β_4 = level change in the control group after the intervention
β_5 = trend change in the control group after the intervention
β_6 = coefficient for the level change in the intervention group
β_7 = coefficient for the trend change in the intervention group
β_8 = coefficient for the impact of GDP on trade
β_9 = coefficient for the impact of visitor numbers on trade
β_{10} = coefficient for the impact of season on trade, categorical variable
$\in$ = error term
